# Role of MNX1-mediated histone modifications and PBX gene family in MNX1-induced leukemogenesis

**DOI:** 10.1038/s41598-026-36367-8

**Published:** 2026-01-19

**Authors:** Eric Malmhäll-Bah, Anders Östlund, Tina Nilsson, Dieter Weichenhan, Marion Bähr, Joschka Hey, Gürcan Tunali, Jenni Adamsson, Susanna Jacobsson, Linda Fogelstrand, Christoph Plass, Lars Palmqvist, Ahmed Waraky

**Affiliations:** 1 Department of Laboratory Medicine, Institute of Biomedicine, University of Gothenburg, Gothenburg, Sweden; 2 Region Västra Götaland, Department of Clinical Chemistry, Sahlgrenska University Hospital, Gothenburg, Sweden; 3 Division of Cancer Epigenomics, German Cancer Research Center (DKFZ), Heidelberg, Germany

**Keywords:** MNX1, PBX, Pediatric AML, Leukemia, t(7;12), Histone methylation, Haematological cancer, Epigenetics

## Abstract

Aberrant MNX1 upregulation is a hallmark of pediatric t(7;12)(q36;p13) AML. In this study, we investigated the downstream targets of MNX1 and their relationship to MNX1-induced histone modifications. Using a comprehensive multi-omics approach combining Tandem Mass Tag–based (TMT) mass spectrometry, RNA-Sequencing, qPCR, antibody-guided chromatin tagmentation sequencing (ACT-Seq), assay for transposase-accessible chromatin using sequencing (ATAC-Seq), and ChIP–qPCR, we identified Pbx1 as a direct MNX1 target and Pbx4 and Pbxip1 as secondary targets associated with leukemic progression. MNX1 binds directly to the Pbx1 promoter, increasing H3K4me3 and reducing H3K27me3 marks, consistent with promoter activation. Despite the transient nature of MNX1 promoter binding, these histone modifications persisted, suggesting a “hit-and-run” epigenetic remodeling mechanism. During leukemic progression, a global increase in H3K4me3 was observed, consistent with broad epigenetic activation. Genome-wide motif analysis further revealed that MNX1 motifs were most enriched within H3K4me1-marked enhancer regions, while ATAC and H3K4me3 enrichment correlated with open chromatin containing PBX family motifs, particularly Pbx4 motifs. These findings suggest that PBX transcription factors might have stage-specific roles in MNX1-driven leukemogenesis, with Pbx1 functioning as an early, MNX1-dependent effector, and Pbx4 and Pbxip1 contributing at later stages through secondary mechanisms. Treatment with the methyltransferase inhibitor Sinefungin, previously shown to block MNX1-mediated histone methylation and leukemogenesis, significantly reduced MNX1-induced Pbx1 expression, consistent with promoter-specific, methylation-dependent regulation at this early preleukemic stage, whereas Pbx4 and Pbxip1 remained unaffected, supporting their roles as secondary targets during leukemic progression.

**Supplementary Information:**

The online version contains supplementary material available at 10.1038/s41598-026-36367-8.

## Introduction

Acute myeloid leukemia (AML) accounts for approximately 15–20% of childhood leukemias^1^. It is characterized by the clonal expansion of hematopoietic stem and progenitor cells (HSPCs), resulting in ineffective hematopoiesis and bone marrow failure^2^. The overall survival rate for AML is notably lower than that of the more common pediatric acute lymphoblastic leukemia (ALL), largely due to various genetic alterations associated with poor prognosis^3^. One such aberration, the translocation t(7;12)(q36;p13), has been identified in children diagnosed under the age of 24 months^4–10^, accounting for approximately 7–30% of infant AML^7,10^ cases and has been included in the 5th edition of the World Health Organization (WHO) Classification of Hematolymphoid Tumors^11^. In all patients with t(7;12), overexpression of *MNX1* has consistently been identified as the most common event^7,9,12^. Using human induced pluripotent stem cells (iPSC), we previously confirmed that this translocation leads to elevated *MNX1* expression^4^, through an enhancer-hijacking mechanism that activates the *MNX1* promoter via enhancers from the *ETV6* locus^6^.

MNX1 is a homeobox transcription factor previously recognized for its role in the development of motor neurons and pancreatic beta cells^13–15^. However, recent studies have implicated MNX1 in oncogenesis, with its involvement observed in AML and in prostate, colorectal, bladder, and hepatocellular cancers^16–21^. In AML, we have shown that MNX1 interacts with methyl transferases and components of the methionine cycle, leading to extensive histone modifications, particularly H3K4me3 and H3K27me3, which in turn result in aberrant gene expression, widespread chromatin alterations, and DNA damage^5^. Notably, these effects were observed only when MNX1 was ectopically expressed in fetal liver–derived hematopoietic cells, but not in adult bone marrow–derived hematopoietic cells, consistent with the pathogenesis of t(7;12)(q36;p13) AML, which occurs almost exclusively in the pediatric setting^5^.

In this study, we used our previously established murine leukemia model generated by transplantation of MNX1-transduced fetal liver (FL) cells^5^ and compared leukemic cells from this model with pre-transplant MNX1-transduced FL cells cultured in vitro (pre-leukemic) or empty-vector–transduced controls. This approach identified *Pbx1* as an MNX1-regulated gene and revealed *Pbxip1* and *Pbx4* as secondary targets associated with leukemic transformation. This expands the potential role of PBX transcription factors in t(7;12) AML. While PBX1 has been extensively studied in leukemia, little is known about the contributions of PBXIP1 and PBX4 in this context^22–25^. PBXIP1, originally described as a PBX1-interacting protein that modulates PBX-HOX complexes^26^, has been linked to erythrocyte differentiation and is upregulated in AML^27^, while PBX4, though less characterized, has been associated with hematopoietic development and may contribute to transcriptional dysregulation in leukemia^24^.

## Results

### Downstream targets of MNX1

**Fig. 1:**
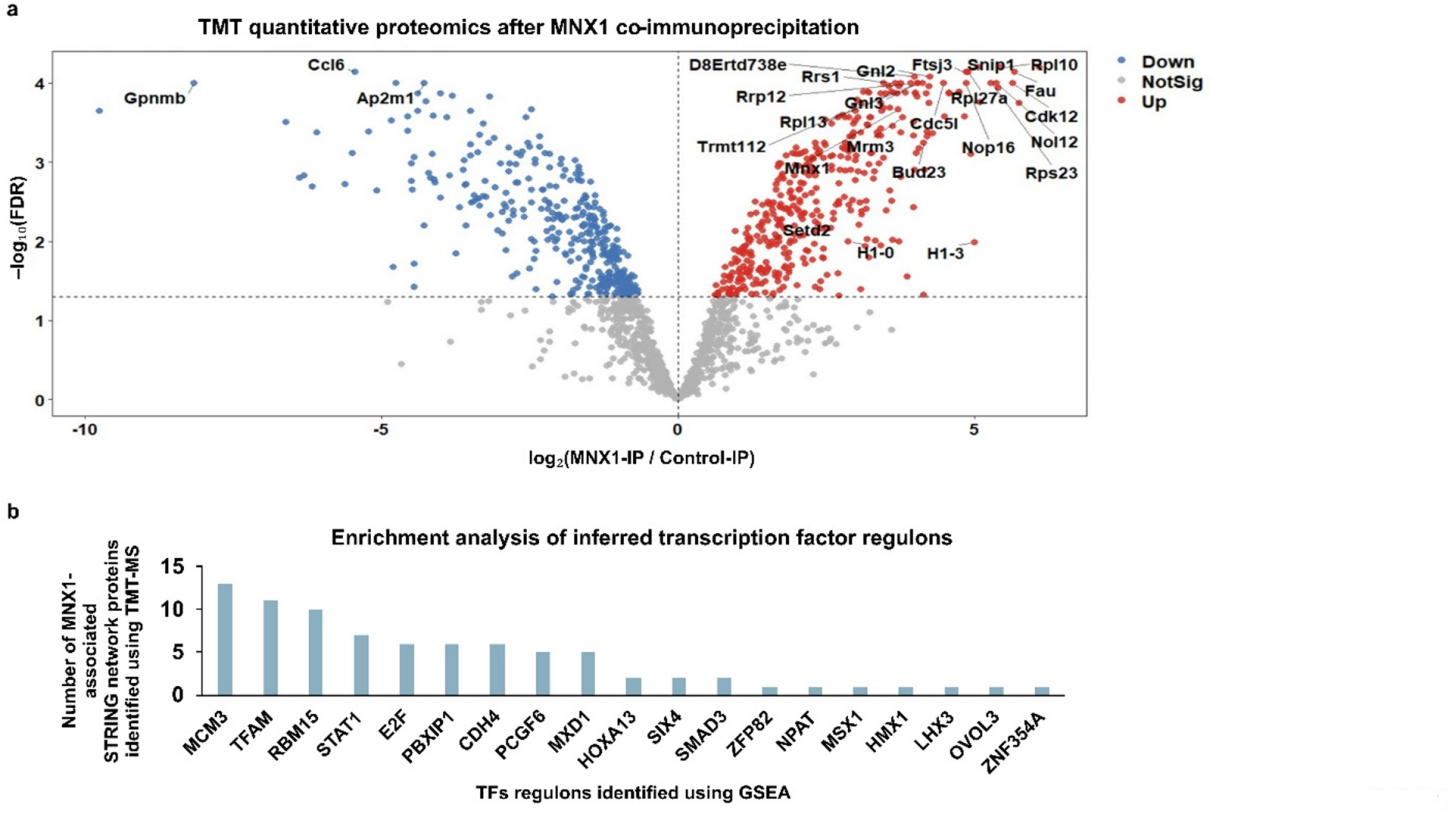
Downstream targets of MNX1. (**a**) Volcano plot showing differential enrichment of proteins identified by TMT-based quantitative proteomics comparing MNX1 co-immunoprecipitation samples with control immunoprecipitations. The x-axis shows the log₂ fold-change log₂(M/C), and the y-axis shows –log₁₀(FDR). Proteins with FDR < 0.05 are considered significantly enriched. Upregulated proteins are shown in red, downregulated in blue, and non-significant proteins in grey. (**b**) Histogram showing the enrichment of inferred transcription-factor regulons, identified by GSEA of transcription-factor target gene sets from MNX1-dependent RNA-Seq data, within the MNX1-associated STRING protein–protein interaction (PPI) network identified by TMT-based co-immunoprecipitation of MNX1. The top 19 enriched regulons are shown.

To identify MNX1 downstream targets, we integrated the MNX1 co-IP/TMT interactome with transcription-factor regulons (transcriptional regulators) enriched in MNX1-dependent RNA-Seq. Regulators that both associate with MNX1 protein network and whose target genes change with MNX1 expression were prioritized as downstream effectors. MNX1-associated protein complexes were identified by co-immunoprecipitation of MNX1 from bone marrow (BM) of mice with MNX1-induced AML, followed by TMT mass spectrometry (see Materials and Methods). As previously described^5^, the leukemia BM (M1-3) were obtained by transplanting NBSGW mice with MNX1 transduced fetal-liver (FL) cells; controls were FL cells transduced with empty vector (C1-3), a schematic overview of the experimental workflow is provided in Supplementary Fig. 1a. TMT analysis identified numerous MNX1-binding partners, including several methyltransferases, ribosomal proteins, spliceosome-associated proteins and histone variants (Fig. 1a; Supplementary Fig. 1b,c; Supplementary Table S3), consistent with our prior findings^5^. MNX1-dependent transcriptional regulators were inferred by gene-set enrichment analysis (GSEA) of transcription-factor target gene sets. We applied GSEA to RNA-Seq from pre-transplantation (*Invitro* FL; Supplementary Fig. 2, 3) and post-transplantation (BM leukemia) MNX1 transduced FL compared to empty vector transduced FL cells (Published data^5^; Supplementary Fig. 3). Using the TMT-identified MNX1-associated proteins, we built a STRING protein–protein interaction (PPI) network (version 12.0; species: *Mus musculus*) with a medium confidence interaction score threshold of 0.4. Active interaction sources included experiments, databases, co-expression, and text mining. The resulting network was analyzed for enrichment of inferred transcriptional regulators, and the top 19 enriched regulons are summarized in Fig. 1b. This analysis highlighted regulators linked to DNA and mitochondrial replication/repair, spliceosome function, and signaling pathways (Fig. 1b). Prominent regulators included mini-chromosome maintenance proteins (Mcm3), RNA Binding Motif Protein 15 (Rbm15), Mitochondrial Transcription Factor 1 (Tfam), Signal Transducer and Activator of Transcription 1(Stat1), E2F family members, and Pbx Homeobox Interacting Protein 1 (Pbxip1). Pbxip1 was of particular interest due to its association with Pbx1, a pioneer transcription factor in the homeobox family, known for its role in development and leukemic transformation^22^. Further STRING analysis of Pbx1 interactome revealed several associations with histone modifiers and methyltransferases identified in our mass spectrometry results, including Kmt2a and Smarca4, as well as with MNX1’s translocation partner Etv6 in t(7;12) AML and several other homeobox proteins (Supplementary Table S7).

**Fig. 2:**
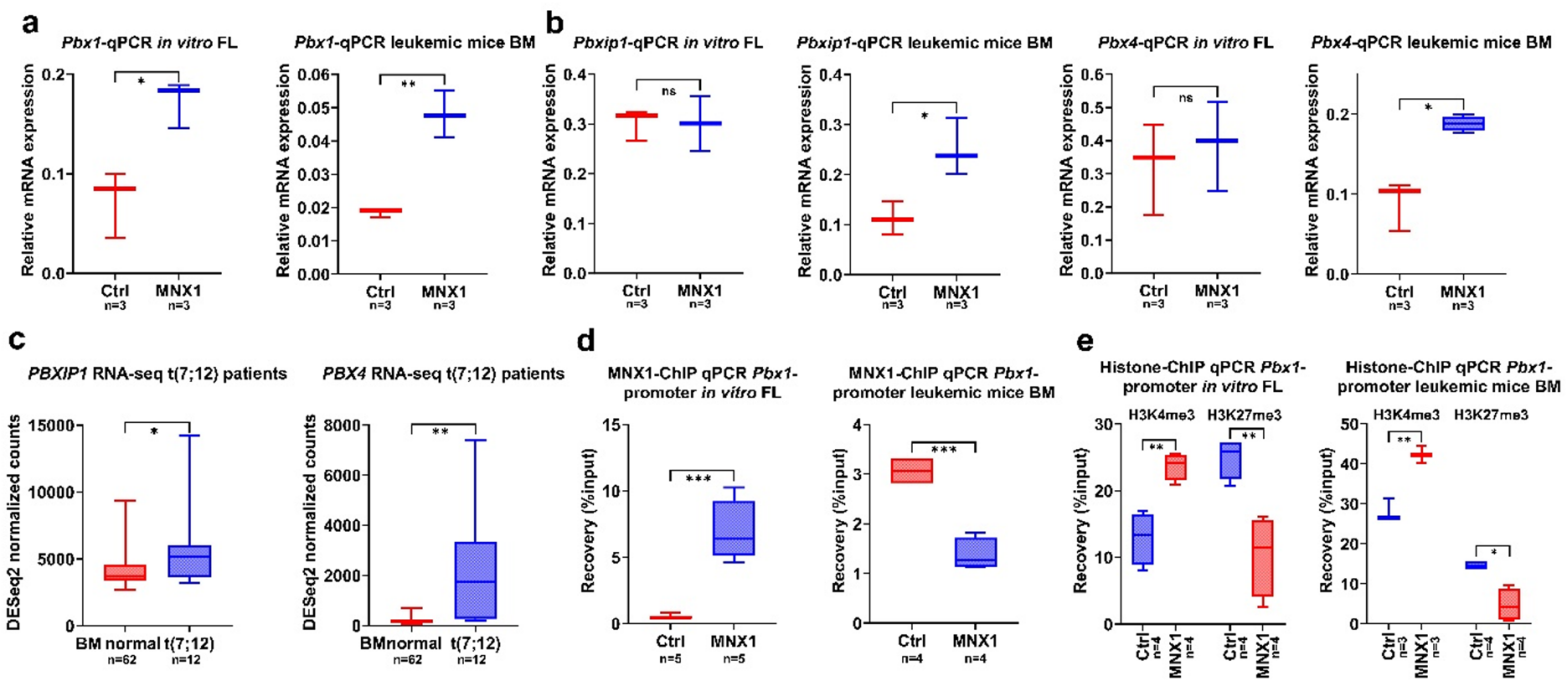
Pbx family genes are upregulated in MNX1-driven pathology. Box-and-whisker plots show median, interquartile range, and full data range of (**a**) Relative expression of *Pbx1*, and (**b**) *Pbx4* and *Pbxip1*, normalized to *Hprt* and measured by qPCR in MNX1-transduced fetal-liver cells versus empty-vector control (Ctrl), and in leukemic bone-marrow cells from MNX1-transplanted mice versus controls. (**c**) DESeq2-normalized expression of PBX4 and PBXIP1 in pediatric AML patient samples carrying t(7;12) compared with normal BM from healthy donors (TARGET AML dataset; Supplementary Table S8). (**d**) MNX1 enrichment, and (**e**) H3K4me3 and H3K27me3 enrichment, at the Pbx1 promoter in FL cells or leukemic BM cells, determined by ChIP-qPCR. Statistical tests: Student’s *t*-test (**a**, **b**, **d**, **e**) or Mann–Whitney U-test (**c**). Significance is indicated as *p* < 0.05 (*), *p* < 0.01 (**), *p* < 0.001 (***). *n* denotes the number of biological replicates.

Using qPCR, we examined *Pbxip1* and *Pbx1* gene expression in pre-transplant in vitro FL cells and leukemic BM from mice. *Pbx1* was upregulated in both settings (Fig. 2a), whereas *Pbxip1* showed increased expression only in leukemic BM (Fig. 2b). Further analysis of other Pbx family members (including *Pbx2* and *Pbx3*) revealed that *Pbx4* was also exclusively upregulated in leukemic BM (Fig. 2b; Supplementary Fig. 4a), similar to *Pbxip1*. These findings were consistent with RNA-Seq data from pediatric t(7;12) AML samples in the COG-NCI TARGET dataset (Supplementary Table S8), where both *PBXIP1* and *PBX4* were upregulated compared to normal BM (Fig. 2c). Across pediatric cytogenetic subtypes in the same dataset, *PBX4* showed the highest median expression in t(7;12) compared with MLL, inv(16), and t(8;21), whereas *PBXIP1* was highest in t(8;21) and second highest in t(7;12) (Supplementary Fig. 4c; Supplementary Table S8). Together, these results suggest that while Pbx1 functions as an early target of MNX1 in preleukemic cells, Pbxip1 and Pbx4 act as secondary (indirect) targets whose altered expression arises with MNX1 leukemia progression.

### H3K4 histone methylation as a persistent marker of MNX1-mediated leukemic progression

Given Pbx1’s potential role as an early target in preleukemic cells, we further examined MNX1’s interaction with the *Pbx1* promoter. We found that MNX1 directly binds to this promoter region (Fig. 2d). This binding was associated with an increase in H3K4me3 (a histone marker for active promoters) and a decrease in H3K27me3 (a histone marker for transcriptional repression) (Fig. 2e). These findings align with our previous observations, which demonstrated a global increase in H3K4me3 and a decrease in H3K27me3 following MNX1 ectopic expression^5^. Interestingly, although *Pbx1* expression remained consistently high in both pre-transplantation in vitro FL cells and leukemic BM, MNX1 occupancy at the Pbx1 promoter was reduced in leukemic BM (Fig. 2d). Despite this reduction, H3K4me3 levels at the *Pbx1* promoter increased slightly, while H3K27me3 levels decreased further in the leukemic BM compared with FL cells (Fig. 2e), suggesting that MNX1-induced histone modifications persist even after its promoter binding diminishes.

**Fig. 3:**
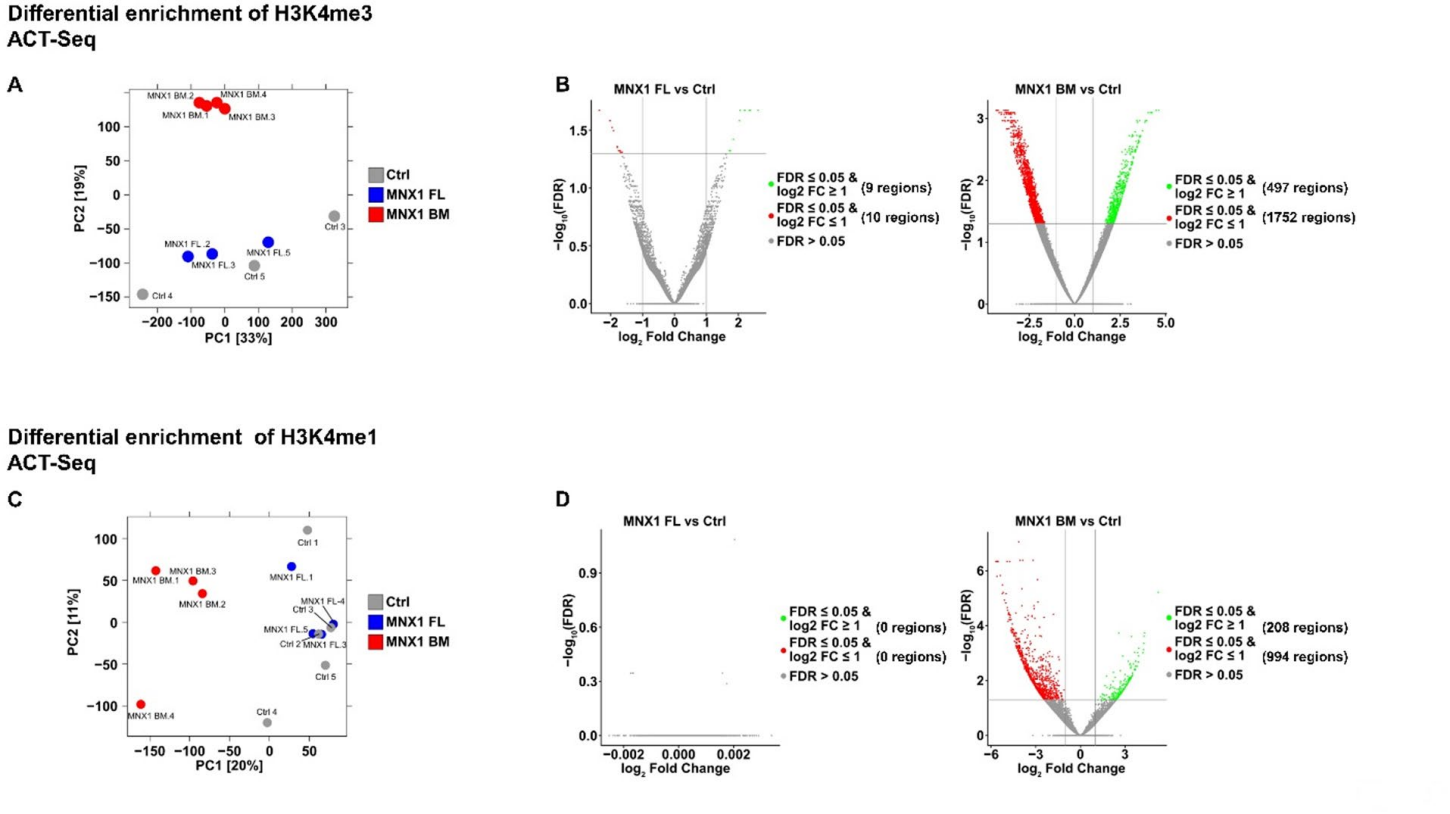
Alterations in histone methylation are associated with leukemia development. (**a** & **c**) Principal component analysis scatter plot of normalized median of ratios using Diffbind and DESeq2 showing variations between the samples in (**a**) H3K4me3 differential enrichment and (**c**) H3K4me1 differential enrichment. (**b** & **d**) Volcano plot depicting the differential enrichment of H3K4me3 (**b**), or H3K4me1 (**d**) between MNX1-vector transduced FL cells (blue) and empty vector transduced FL cells (Ctrl-grey; left panel) or mice leukemia BM cells (red) and empty vector transduced FL cells (right panel).

To further examine the H3K4me3 histone modifications genome-wide, we performed ACT-Seq for both H3K4me3 and H3K4me1 (an active-enhancer mark) in MNX1-transduced in vitro FL cells and in leukemic BM (Fig. 3). Principal component analysis (PCA) revealed minimal differences between MNX1-transduced FL and control cells but marked divergence between leukemic BM and controls (Fig. 3a,c). Correspondingly, differential enrichment analysis showed significant global increases in H3K4me3/H3K4me1 in leukemic BM relative to control cells, whereas no significant differences were observed in pre-transplant FL cells (Fig. 3b,d). The increase in H3K4me3 levels during leukemic development was further supported by GSEA analysis of RNA-Seq data, which showed a higher enrichment of differentially expressed genes associated with H3K4 methylation in leukemic BM compared to FL cells (Supplementary Fig. 5), indicating broad activation of this epigenetic program during leukemic progression.

Annotation of the differentially enriched regions confirmed expected distributions, with H3K4me3 localized mainly to promoter regions (~ 70%) and H3K4me1 predominantly at distal intergenic and intronic regions (~ 60%; Supplementary Fig. 6). Functional analysis of these promoter-associated regions linked them to genes involved in myeloid differentiation, senescence, cell-cycle regulation, stem-cell maintenance, and mRNA/proteasome catabolic processes (Supplementary Fig. 7; Supplementary Tables S9-S10), pathways consistent with those enriched in our previous MNX1-associated RNA-Seq and proteomic analyses^5^.

### Pbx motifs show high enrichment in regions marked by MNX1-ATAC and H3K4 peaks

Given that MNX1 directly activates Pbx1 and that Pbx4 becomes overexpressed during leukemic progression, coinciding with global increases in H3K4 methylation, we next investigated whether Pbx family transcription factors contribute to MNX1-induced epigenetic remodeling.

**Fig. 4:**
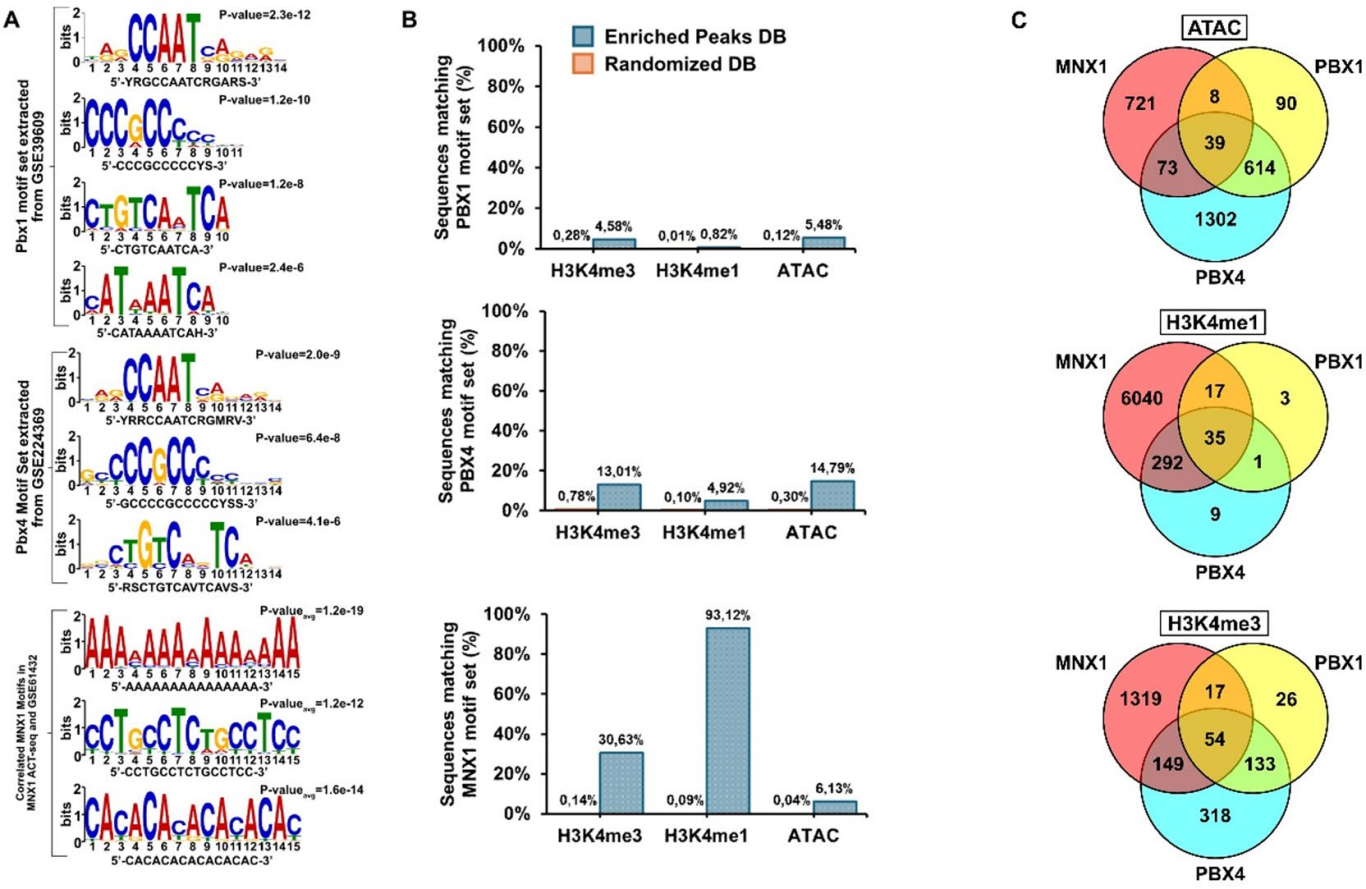
Pbx motifs are enriched in MNX1 peaks corresponding to pathways mediated by MNX1. (**a**) Enrichment analysis using MEME-suite’s STREME-function identifies Pbx1, Pbx4 and MNX1 transcription factor motifs overrepresented at differentially accessible promoters and enhancers from ChIP-seq. For a complete list of enriched motifs identified in each condition and genomic region, refer to Supplementary table S4, S5 & S6. (**b**) Histogram showing the enrichment of Pbx1 (upper panel), Pbx4 (middle panel) and MNX1(lower panel) motifs identified within MNX1 peaks derived from differentially accessible peaks of ATAC-Seq, as well as differential enrichment peaks for H3K4me3 and H3K4me1. Randomly generated sequences matched for equal size, GC content, and sequence length distribution were used as negative control (Randomized DB). (**c**) Venn diagram showing the overlap between MNX1, Pbx1 and Pbx4 motif enrichment within ATAC, H3K4me1 and H3K4me3 peaks.

To define their DNA-binding motifs, we identified Pbx1 and Pbx4 motifs from publicly available ChIP-seq datasets—mouse embryonic trunk for Pbx1 (GSE39609) and mouse testis for Pbx4 (GSE224369) using the MEME-suite pipeline (Fig. 4a; Supplementary Tables S4–S5). These motifs were then tested for enrichment within MNX1-associated H3K4me3, H3K4me1, and ATAC-Seq peaks derived from leukemic bone marrow (BM) samples using MAST, as described in the Materials and Methods.

This analysis revealed a significant enrichment of Pbx4 motifs, particularly within ATAC-Seq and H3K4me3 peaks, with motif frequencies of approximately 10–15%. Pbx1 motifs were also enriched but at lower frequencies (Fig. 4b). To compare these distributions with MNX1 itself, we next identified MNX1-binding motifs using ACT-Seq data from leukemic BM. Although reproducibility of individual MNX1 ACT-Seq peaks across replicates was moderate (Supplementary Fig. 8–9), the derived motifs were highly consistent among replicates and closely matched published MNX1 ChIP-seq datasets (GSE61432) (Fig. 4a; Supplementary Fig. 10).

Interestingly, the MNX1 motifs identified here differed from the canonical MNX1 motif previously described using the Genomatix platform^28^. When we reanalyzed those canonical motifs, they were also enriched within MNX1 ACT-Seq peaks as well as within ATAC, H3K4me1, and H3K4me3 regions (Supplementary Fig. 11). However, their frequencies did not exceed those observed in GC-matched randomized controls, whereas the newly identified MNX1 motifs showed significantly higher enrichment relative to random sequences (Supplementary Fig. 11), suggesting that they better represent MNX1’s binding specificity in leukemic cells.

Genome-wide, MNX1 motifs were most abundant within H3K4me1-marked enhancer peak regions (~ 93% of sites containing the motif) and less frequent within H3K4me3 and ATAC-Seq promoter peaks (Fig. 4b; Supplementary Table S6). This distribution suggests that MNX1 primarily acts through enhancer-associated chromatin and that part of its transcriptional output may be mediated by downstream co-regulators such as the Pbx family.

To explore this possibility, we compared motif co-localization among MNX1, Pbx4, and Pbx1 across ATAC, H3K4me3, and H3K4me1 peaks. The overlap between MNX1–Pbx4 and MNX1–Pbx1 motifs was modest, indicating that Pbx4, and to a lesser extent Pbx1, occupy partially distinct genomic regions influenced by MNX1-induced epigenetic changes specifically, regions of open chromatin (ATAC-Seq peaks) and active promoters (H3K4me3 peaks) (Fig. 4c).

**Fig. 5:**
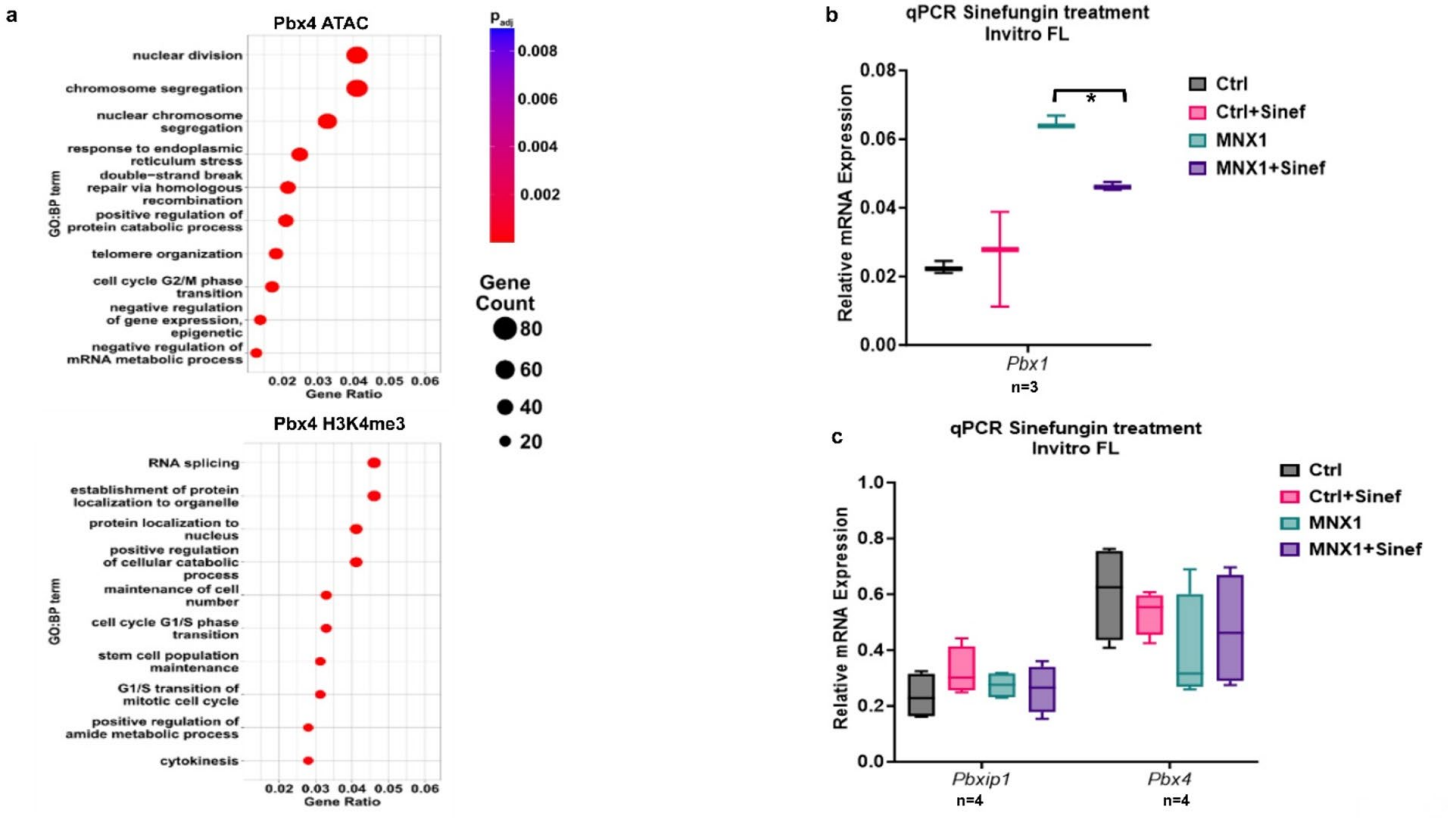
Pathway enrichment and methyltransferase inhibition effects on Pbx family. (**a**) Bubble plots showing pathway enrichment for Pbx4 motifs identified within MNX1-regulated regions from ATAC-Seq (upper panel) and H3K4me3 ACT-Seq (lower panel), generated using the ClusterProfiler package. Bubble size reflects the number of genes associated with each pathway, and bubble color indicates the adjusted p-value. (**b–c**) Box-and-whisker plots showing median, interquartile range, and full data range of relative mRNA expression for Pbx1 (**b**) and Pbxip1 and Pbx4 (**c**), normalized to Hprt as a loading control. Expression levels were measured in vitro fetal-liver (FL) cells transduced with either MNX1 or empty vector (Ctrl), and treated with vehicle or 5 µM Sinefungin (Ctrl + S, MNX1 + S). Fresh Sinefungin was added daily for one week following viral transduction. Tukey’s multiple comparisons test was used for post-hoc analysis. Significance is indicated as *p* < 0.05 (*). n represents biological replicates.

Pathway enrichment analysis of promoter regions containing Pbx4 and Pbx1 motifs within ATAC-Seq and H3K4me3 peaks identified biological processes previously linked to MNX1 function^5^, including chromosome segregation, erythroid differentiation, cell-cycle G2/M transition, telomere organization, epigenetic regulation of transcription, stem-cell maintenance, and DNA double-strand break repair (Fig. 5a; Supplementary Fig. 12).

Together, these findings may suggest that MNX1 primarily remodels enhancer-associated chromatin through H3K4 methylation and directly activates Pbx1, while Pbx4 and possibly Pbxip1 are engaged later as secondary transcriptional regulators, contributing to the maintenance of MNX1-driven gene programs during leukemic progression.

To validate the early role of Pbx1 as a direct downstream target of MNX1-induced epigenetic modification, and the secondary roles of Pbx4 and Pbxip1 that emerge only during leukemic progression, we treated preleukemic MNX1-transduced FL cells with the pan-methyltransferase inhibitor Sinefungin, previously shown to block MNX1-mediated histone methylation and leukemia induction^5^. Sinefungin treatment significantly reduced MNX1-induced Pbx1 expression (Fig. 5b), consistent with methylation-dependent regulation of Pbx1 at this early stage. In contrast, Pbx4 and Pbxip1 expression levels were unaffected (Fig. 5c), supporting their classification as secondary, indirectly regulated targets that become more relevant during leukemic progression.

## Discussion

In this study, we aimed to elucidate the downstream targets by which MNX1, a pivotal factor in t(7;12) pediatric AML, drives chromatin and histone modifications. Our findings demonstrate the role of MNX1 in upregulating *Pbxip1*,* Pbx1*, and *Pbx4*, highlighting their possible contributions to MNX1-induced epigenetic alterations, and positioning them as potential therapeutic targets for t(7;12) AML.

Recently, we have shown that MNX1 activates transcription by interacting with epigenetic modifiers, including histone methyltransferases, which alter the chromatin landscape and regulate gene expression^5^. Consistent with these findings, the current study further reinforces MNX1’s role in transcriptional activation through epigenetic modification. We observed that MNX1 binding to the *Pbx1* promoter is associated with increased H3K4me3 and reduced H3K27me3, two key epigenetic marks that regulate gene activation and repression, respectively. MNX1’s association with histone-modifying enzymes was further supported by TMT-based quantitative mass spectrometry of MNX1 co-immunoprecipitates, which identified multiple methyltransferases and histone variants in complex with MNX1. The significance of H3K4me3 is particularly noteworthy, as alterations in this histone mark have been linked to the regulation of oncogene expression and leukemic stem cell maintenance^29^. In MLL-rearranged AML, changes in H3K4me3 levels influence leukemic stem cell persistence and activation of oncogenes such as HOXA9 and MEIS1^29^. Additionally H3K4me1, often associated with active enhancers, and H3K4me3 have been associated with MEIS1 binding sites, another member of the homeobox family of transcription factors, during embryological development^30^, and were linked to the regulation of gene networks involved in myeloid differentiation^31^, an important pathway which we have shown to be induced by *MNX1* ectopic expression^5^.

An intriguing aspect of our data is the transient nature of MNX1 occupancy at the *Pbx1* promoter: MNX1 binding is evident in MNX1-transduced fetal liver cells prior to transplantation but diminishes in leukemic bone marrow, while the associated histone modifications, particularly H3K4me3, persist. This pattern suggests a ‘hit-and-run’ mechanism for *Pbx1*, in which MNX1 initiates chromatin remodeling but is no longer required to maintain it. This concept is similar to other histone methylation recruiters and writers, such as PRC2 for H3K27me3 methylation and SET1 for H3K4me3 methylation, where histone marks persist long after the dissociation of the methyltransferases^32,33^. The sustained histone modifications imply that early MNX1 interactions have long-term consequences in t(7;12) AML, where MNX1 initiates leukemogenesis but may not need to persist for the disease to progress. Moreover, the transient nature of MNX1’s binding could help explain the low reproducibility we observed with MNX1 peaks in ACT-Seq, further suggesting that MNX1’s role may primarily be to “mark” key chromatin regions before dissociating. It is also worth noting that MNX1 chromatin profiling was performed using ACT-Seq rather than traditional ChIP-seq, which is reported to produce non-specific cleavage of accessible regions^34^.

The identification of *Pbx1* as a direct transcriptional target of MNX1, and of *Pbx4* and *Pbxip1* as secondary targets associated with MNX1-driven leukemic progression, further builds on existing literature regarding the PBX family’s role in hematopoiesis and leukemic transformation^22–24^. PBX1, in particular, has been well-characterized as a partner of HOXA9, collaborating to promote leukemogenesis by activating critical genes involved in cell survival and proliferation^25^. However, PBXIP1 and PBX4 are less extensively studied in the context of leukemia. Beyond PBXIP1’s established role as an interacting partner with PBX1, PBX2, and PBX3, blocking the recruitment of PBX-HOX heterodimers and inhibiting the transcriptional activity of PBX^26^, recent study has suggested a potential role for PBXIP1 in erythrocyte differentiation^27^, and elevated PBXIP1 levels have been observed in AML using Oncomine datasets^23^. PBX4 has been implicated in regulating key developmental pathways in hematopoiesis^24^ that may drive malignant transformation by altering cell differentiation and proliferation processes. This warrants further investigation into the specific contributions of PBXIP1 and PBX4 to MNX1-driven leukemia and their potential as therapeutic targets.

The enrichment of PBX family binding motifs in MNX1-induced H3K4me1, H3K4me3 and ATAC-Seq peaks suggests a possible cooperative relationship between MNX1 and the PBX transcriptional network in driving chromatin remodeling as downstream effectors of MNX1. This interaction aligns with findings in MLL-rearranged AML, where PBX1 engages with chromatin-modifying complexes to regulate gene expression^35^. Given that histone methylation plays a crucial role in AML progression, these epigenetic modifications may represent promising therapeutic targets. Methyltransferase inhibitors, such as DOT1L inhibitors, have shown preclinical efficacy in models of MLL-rearranged AML^36,37^. In our study, the broad-spectrum methyltransferase inhibitor Sinefungin, previously shown to inhibit the MNX1-drived leukemogenesis^5^, was able to downregulate MNX1-induced *PBX1* expression, reinforcing the potential of targeting histone methylation in t(7;12) AML. However, the expression of *Pbx4* and *Pbxip1* was not significantly affected by Sinefungin treatment, possibly due to their later induction during leukemia development. This temporal regulation suggests that PBX4 and PBXIP1 may play roles in maintaining the leukemic state rather than initiating it, highlighting the complexity of MNX1’s downstream effects.

In conclusion, our study provides new insights into the MNX1 signaling pathway shedding light on the interplay between MNX1, chromatin regulation, and the PBX family. These findings lay the groundwork for further investigation into the timing and context of PBX1, PBX4 and PBXIP1 involvement in leukemogenesis and their response to methyltransferase inhibition, offering a promising approach to halting or reversing leukemic progression in t(7;12) AML.

## Methods

### Plasmids

The human MNX1 expression vector was constructed using the MSCV Retroviral Expression System (Takara Bio, Cat. No. 634401), which enables stable gene expression under the control of the viral LTR promoter. The human MNX1 coding sequence (Supplementary Table S1) was modified to include an N-terminal HA-tag (36 bp), followed by a 24 bp linker sequence, and the first ATG codon was removed to prevent unintended translation initiation. The whole modified MNX1 gene with flanking restriction sites was synthetized at Integrated DNA Technologies and cloned into pMSCV-IRES-GFP and pMSCV-IRES-YFP vectors after digestion with the corresponding restriction enzymes.

### Animal model and cell cultures

Mice were generated and kept at the Gothenburg University Laboratory for Experimental Biomedicine Animal Facility (Gothenburg, Sweden) in an aseptic environment. All animal experiments were approved by the Swedish Board of Agriculture (Jordbruksverket) and the Gothenburg Animal Ethics Committee (permit number Dnr 5.8.18–17008/2021, approved on 27 October 2021). All experimental procedures were performed in accordance with relevant institutional, national, and international guidelines and regulations, including the Swedish Animal Welfare Act, EU Directive 2010/63/EU for animal experiments, and are reported in compliance with the ARRIVE guidelines.

Bone marrow (BM) cell lines (In vitro FL) transduced with human *MNX1* or empty vector control were established as previously detailed in^5^. Briefly, 8–12-week-old C57Bl/6 mice (Charles River Laboratories Inc., Wilmington, MA, USA) were used for mating, and fetal livers (FL) were extracted from embryos at embryonic day E14.5. Whole fetal liver (FL) hematopoietic cells were maintained in culture (DMEM (Merck Millipore, Darmstadt, Germany), supplemented with 18% fetal calf serum 10 ng/mL human interleukin-6 (Peprotech, Cranbury, NJ, USA), 6 ng/mL murine IL-3 (Peprotech), and 50 ng/mL murine stem cell factor (Peprotech)) until transduction with *MNX1* or empty vector control. Cells were transduced by cultivation on irradiated E86 producers for 2 days in the presence of 5 µg/mL protamine sulfate (Merck Millipore). FACS-sorted (FACSAria, BD Biosciences, Franklin Lakes, NJ, USA) GFP + and/or YFP + cells were maintained in culture 5–7 days post-transduction prior to transplantation into 8–12-week-old male NOD.Cg-*Kit*^*W−41 J*^ *Tyr*^*+*^ *Prkdc*^*scid*^ *Il2rg*^*tm1Wjl*^/ThomJ (NBSGW) mice (The Jackson Laboratory, Bar Harbor, ME, USA). 0.8 × 10^6^ transduced cells were transplanted via tail vein injections in two experimental arms *MNX1* or empty vector control. Engraftment was monitored by FACS-analysis of GFP + and/or YFP + expression in peripheral blood every 2 weeks. Each experimental arm consisted of 4–6 mice and was repeated 3 biological times. The number of mice was chosen based on previous studies in similar experimental setups, where this number provides sufficient power to detect significant differences while minimizing animal use. Randomization was used to allocate experimental units to control and *MNX1* groups, and the order of measurements was randomized to minimize potential confounders, such as cage location.

Exclusion criteria included the removal of animals that suffered injuries due to fighting between mice, which could affect the validity of the results. If any animal exhibited signs of severe injury, it was excluded from the study, and the reason for exclusion was recorded.

From each biological replicate, one or two bone marrow (BM) samples were used for PCR, ChIP-qPCR, and ACT-Seq analysis. Blinding was applied during the peripheral blood assessments every 2 weeks, as well as during the collection of BM samples for PCR, ChIP-qPCR, and ACT-Seq analysis, to minimize any potential bias in outcome assessment.

Animals were euthanized using the isoflurane (Baxter, Deerfield, IL, USA) drop jar method followed by cervical dislocation. A sealed chamber was prepared by placing a gauze pad soaked with 1.7% isoflurane and the chamber was tightly sealed to prevent the escape of vapors. Upon the onset of deep anesthesia, indicated by the absence of the righting reflex and loss of pedal withdrawal reflex, cervical dislocation was performed manually as per established guidelines. Blood counts were determined on a Sysmex KX-21 Hematology Analyzer (Sysmex, Norderstedt, Germany).

### Immunoprecipitation and tandem mass tag–based (TMT) mass spectrometry

Anti HA-magnetic beads from Thermo Fisher Scientific (catalog no. 88836) were used for immunoprecipitation. After blocking with 0.1% BSA in PBS, 1 mg of cell lysates were incubated with HA-magnetic beads for 1 h at RT. The immune complexes were washed three times with modified RIPA lysis buffer (0.05 M Tris-HCl (pH 7.5), 1% NP40, 0.25% Sodium Deoxycholate, 150 mM NaCl, 1 mM EDTA) and eluted with 2% SDS in 50mM Tris-HCL with 1mM EDTA (Invitrogen).

The samples were then digested with trypsin using modified filter-aided sample preparation (FASP) method^38^. Samples were reduced in 100 mM dithiothreitol at 60 °C for 30 min, transferred to Microcon-30 kDa Centrifugal Filter Units (Merck), washed with 8 M urea and digestion buffer (50 mM TEAB, 0.5% sodium deoxycholate (SDC)) prior to alkylation with 10 mM methyl methanethiosulfonate for 30 min at room temperature. Proteins were digested in two steps with trypsin (Pierce MS grade Trypsin, Thermo Fisher Scientific) at 37 °C at a final enzyme to protein ratio of 1:50. Peptides were collected by centrifugation and labelled using TMT10plex isobaric label reagents (Thermo Fisher Scientific) according to the manufacturer´s instructions. The individually labelled samples were pooled to one TMT-set and purified using HiPPR detergent removal kit (Thermo Fisher Scientific). SDC was removed by acidification with 10% trifluoroacetic acid. The TMT-set was fractionated to five fractions using Pierce High pH Reversed-Phase Peptide Fractionation columns (Thermo Fisher Scientific) and acetonitrile (ACN) as organic phase. The fractions were evaporated to dryness and reconstituted in 3% ACN, 0.2% formic acid (FA) for LC-MS analysis.

The fractions were analyzed on an Orbitrap Fusion Lumos Tribrid mass spectrometer equipped with a FAIMS-Pro ion mobility system and interfaced with an Easy-nLC1200 liquid chromatography system (all Thermo Fisher Scientific). Peptides were trapped on an Acclaim Pepmap 100 C18 trap column (100 μm x 2 cm, particle size 5 μm, Thermo Fisher Scientific) and separated on an in-house packed analytical column (38 cm x 75 μm, particle size 3 μm, Reprosil-Pur C18, Dr. Maisch) using a linear gradient from 5% to 28% ACN in 0.2% FA over 77 min at a flow of 300 nL/min. FAIMS Pro was alternating between compensation voltages (CVs) of −40, −60 and − 80, with the same data-dependent settings for all three CVs. The precursor ion mass spectra were acquired at a resolution of 120 000 and an m/z range of 375–1375. The top eight most abundant precursors with charges 2–7 were isolated with an m/z window of 0.7 and fragmented by collision induced dissociation (CID) at 35%. Fragment spectra were recorded in the ion trap at Rapid scan rate. The ten most abundant MS2 fragment ions were isolated using multi-notch isolation for further MS3 fractionation. MS3 fractionation was performed using higher-energy collision dissociation (HCD) at 65% and the MS3 spectra were recorded in the Orbitrap at 50 000 resolution and an m/z range of 100–500.

Data analysis was performed with Proteome Discoverer (Version 2.4, Thermo Fisher Scientific) using Mascot (Version 2.5.1, Matrix Science) as a search engine. The data was matched against the reviewed swissprot mouse database (May 2020; 17116 entries). Tryptic peptides were accepted with 1 missed cleavage; methionine oxidation was set as variable modification and cysteine methylthiolation, TMT10plex were set as fixed modifications. Precursor mass tolerance was set to 5 ppm and fragment mass tolerance to 0.6 Da. Percolator was used for PSM validation with a strict FDR threshold of 1%. For reporter ion quantification peak integration was set to most confident centroid and a tolerance of 3 mmu. Only unique peptides were used for protein quantification (Supplementary Table S3).

For quantitative and statistical analysis, protein abundances from MNX1-immunoprecipitated samples (M1–M3) and control immunoprecipitations (C1, C2, C4) were log₂-transformed and, if required, median-normalized per channel. Proteins with at least one valid value in both groups were retained. Differential enrichment was assessed using the *limma* moderated t-test with empirical Bayes shrinkage (*trend = TRUE*), and *p*-values were adjusted for multiple testing using the Benjamini–Hochberg FDR method. Volcano plots depict log₂(M/C) on the *x*-axis and –log₁₀(FDR) on the *y*-axis. Figures were generated in R (v4.x) using *ggplot2* and *ggrepel*. Proteins with FDR < 0.05 were considered significantly enriched.

### Gene expression analysis and next generation sequencing

Total RNA was isolated using the miRNeasy Plus micro kit (Qiagen) and cDNA synthesized with SuperScript III First-Strand Synthesis SuperMix for qRT-PCR (Invitrogen/Thermo Fisher Scientific). TaqMan Gene Expression Assays and TaqMan Universal MasterMix II (Applied Biosystems/Thermo Fisher Scientific) were used for probe-based qPCR assays, listed in Supplementary Table S2. Hprt was used as reference gene for normalization of the expression.

RNA-Seq was performed as described previously in ^5^. Briefly, total RNA was extracted from BM cells from mice with MNX1 induced leukemia and from transduced FL cells after one week of transduction as control. Library preparation and sequencing was carried out by Beijing Genomics Institute. Reads were mapped to the mm10 mouse reference genome using STAR. Differential gene expression was performed using DESeq2 with inclusion criteria of false discovery rate (FDR) less than 0.05 and log2 fold change above |1|. Gene Set Enrichment Analysis^39^ (GSEA, Broad Institute) was used with the Gene Transcription Regulation Database (GTRD) transcription factor targets and legacy transcription factor gene set collection. A pathway in GSEA analysis was regarded significant at nominal p-value < 0.05. Additionally, RNA-Seq data of twelve t(7;12), 62 normal human BM, 46 t(8;21), 42 inv(16) and 47 MLL patients (patient case IDs listed Supplementary Table S8) were obtained from the Children’s Oncology Group (COG)–National Cancer Institute (NCI) TARGET AML initiative data set (available at https://target-data.nci.nih.gov/Public/AML/mRNA-Seq/) and processed as above.

### ChIP-qPCR

ChIP-qPCR was performed as described previously in^5^. In short, iDeal ChIP-qPCR kit (C01010180, Diagenode*)* was used to perform the ChIP assay. Crosslinking was achieved by incubating cells with formaldehyde for 10–15 min at room temperature (RT). Subsequently, the reaction was quenched by the addition of glycine and further incubation for 5–10 min at RT. The cell pellet was then washed with cold PBS and lysed according to manufacturer specifications. DNA was sheared by sonication using a bioruptor for 20 min in 30 s intervals achieving DNA sizes in the 200–600 bp range. Immunoprecipitation was then performed using the antibodies against HA (cell signaling_3724S), H3K27me3 (Diagenode_C15410195, RRID: AB_2753161), H3K4me3 (Diagenode_C15410003, RRID: AB_2924768), and polyclonal rabbit anti-IgG (Diagenode_C15410206, RRID: AB_2722554) as negative control. DNA was purified and, qPCR was performed using SYBR green chemistry and in-house designed primers shown in Supplementary Table S2. The calculation of enrichment was normalized to the respective inputs and done according to the following formulas:

- The % of IP to input formula: % recovery = 2^[(Ct input-log2(5%)) – Ct sample] * 100%.
- The enrichment was calculated as: %recovery IP/% recovery IgG.

Negative control was achieved by immunoprecipitation against HA antibody, followed by detection of random region with low CpG islands and promoter binding density corresponding to chr1:168430579–168,430,704. This region lies at the 5′ end of the Pbx1 locus, approximately 100–200 bp upstream of the transcription start site (TSS) for Pbx1. Designed primers for the random region is shown in Supplementary Table S2.

### RNA-Seq & mass spec integrative analysis

GTRD transcription factor targets gene set from gene set enrichment analysis (GSEA) was used to identify the transcription factor targets from the normalized counts of RNA-Seq. Protein network of the identified proteins from tandem mass spectrometry tag (TMT) was obtained from STRING database (version 12.0; species: *Mus musculus*) with a medium confidence interaction score threshold of 0.4. Active interaction sources included experiments, databases, co-expression, and text mining.

### ATAC and ACT sequencing

Assay for Transposase-Accessible Chromatin (ATAC-Seq) and Active Chromatin Targeting (ACT-Seq) were performed as described previously in^5^. Briefly, bone marrow cells from MNX1-induced leukemic mice, non-transplanted mouse fetal liver (FL) cells containing MNX1-vector as well as FL cells with empty vector were lysed using in resuspension buffer containing 0.1% of NP40 and Tween-20 and 0.01% digitonin. Lysed cells were resuspended in transposition buffer (10 mM Tris-acetate, pH 7.6, 5 mM MgCl2, 10% dimethylformamide, 30% PBS, 0.01% digitonin and 0.1% Tween-20) together with Tn5 transposome and tagmentation were allowed to proceed for 30 min at 37 °C under agitation. For ACT-Seq, targeting antibodies used for the pA-Tn5 transposome-antibody complexes were directed against H3K4me3 (Abcam_ab8580, RRID: AB_306649), H3K4me1 (Abcam_ab8895, RRID: AB_306847), HA-tag (Sigma_H6908, RRID: AB_260070), IgG (Merck Millipore PP64B, RRID: AB_97852) and yeast histone H2B (Hölzel Diagnostika, M30930, Cologne, Germany, RRID: AB_2924769), the latter complexes being used for spike-in to enable sequence read normalization between biological replicates. DNA was purified and checked for quality followed by sequencing on a Illumina next-seq. Reads were trimmed using Trim Galore in conjunction with Cutadapt (v. 1.14) with the options –paired”, “–nextera”, “– length_1 35”, and “–length_2 35. Trimmed reads were mapped against mm10 reference genome using Bowtie2^40^ (v. 2.5.2). Conflicting mapped reads with a phred score lower than 20 were removed using SAMTools^41^ (v. 1.19). Finally, read ends were shifted to center of the transposition event.

HA-tagged MNX1, H3K4me3 and H3K4me1 peaks were retrieved with GoPeaks^42^ (v 1.0.0), using a q-value cut-off of 0.05 and the --broad and --mdist 3000 options for the latter. ATAC-Seq peaks were called using MACS2^43^ with the options --broad, --keep-dup all, --nomodel and a q-value cutoff of 0.05. Differential enrichment analysis was determined using Diffbind^44^ and DESeq2^45^ (v. 3.12) R (v. 4.3.2) package with regions considered differentially accessible or enriched if fulfilling FDR less than 0.05 and a fold change larger than |1|. Peak reproducibility between replicates was determined by Jaccard statistic, using the bedtools jaccard function. Consensus peaks were constructed of replicates using BEDTools^46^ (v. 2.30.0) multiinter function only retaining regions/peaks found in all replicates. Subsequently, regions within the 1 kbp range were merged using the BEDTools merge function. To determine where open-chromatin intersected with histone depositions, BEDTools intersect function was used.

Peak annotation was performed using ChIPseeker^47^ (v. 1.38.0) R package, mapping peaks against TxDb UCSC mm10 known gene^48^ reference and defining the promoter region as ± 3 kbp from transcription starting sites. Promoter regions were used to find enriched GO biological pathways using clusterProfiler^49^ (v. 4.10.0) R package.

### Pbx motif analysis

ChIP-seq datasets of Pbx1 in mouse embryonic trunks^50^ (GSE39609) and Pbx4 in mouse testis^51^ (GSE224369) were retrieved from Gene Expression Omnibus (GEO). Sequencing data in FASTQ format were aligned to mm10 reference genome and converted to BAM-file format using Bowtie2 (v. 2.5.2) and SAMTools. Multi-mapped, unmapped and duplicate reads were filtered out using Sambamba (v. 0.7.1). Peaks were retrieved with MACS2 with a q-value of 0.05. When replicates were available, consensus peaks were constructed of replicate peak sets as described in the ATAC and ACT sequencing section. DNA-binding motifs and their enrichment were analyzed using MEME-Suite (v. 5.5.1). Motifs were retrieved from the Pbx ChIP-seq peak regions with the STREME^52^ function using default parameters, motif minimum and maximum length of 8 and 15, respectively, and binomial test against a random test set consisting 10% of the input sequences to evaluate statistical significance. Subsequently, the top scoring motifs, matching > 10% of peaks in each of the Pbx ChIP-seq data sets were used as a motif sets for a MAST^53^ scan of the consensus peak sets derived from epigenetic data of the MNX1-induced leukemic mice. A positional p-value of < 0.0001 for a motif was deemed as a match and a E-value (combined positionally p-value for all matches within a sequence times the number of sequences in the database) of > 10 of was used as criteria to consider the sequence significant. The resulting motif-matches of the MAST scan are shown Supplementary Table S4 & S5 for the Pbx1 and Pbx4 motif-sets, respectively. As a control the motif-sets were tested against randomly generated sequences produced with the online tool FaBox^54^, recapitulating number of sequences, sequence size distribution and GC-content of the enriched peak data sets. Regions enriched in Pbx-motifs were submitted to pathway enrichment analysis using Clusterprofiler removing redundancy and filtering for pathways by GO level using the simplify function resulting in the most specific pathways.

### MNX1 motif analysis

MNX1 motifs were identified using the STREME function within MEME-suite on each MNX1 ACT-Seq peak set individually, as well as on a publicly available ChIP-seq dataset of Mnx1 in mouse insulinoma cells^28^ (GSE61432), applying parameters consistent with those used in the Pbx motif analysis. The top 10 motifs identified were then assessed for correlation using the MAST function’s built-in correlation feature within MEME-suite; motifs were considered correlated if their average Pearson correlation exceeded 0.8. Correlated motifs found across all peak sets were subsequently analyzed for enrichment in epigenetic data from MNX1-induced leukemic mice, as outlined in the Pbx motif analysis. The motif matches from the MAST scan for the MNX1 motif set are presented in Supplementary Table S6.

## Supplementary Information

Below is the link to the electronic supplementary material.

Supplementary Material 1

Supplementary Material 2

Supplementary Material 3

Supplementary Material 4

Supplementary Material 5

Supplementary Material 6

Supplementary Material 7

Supplementary Material 8

Supplementary Material 9

Supplementary Material 10

Supplementary Material 11

## Data Availability

The datasets generated for this study can be found in the Gene Expression Omnibus (GEO; https://www.ncbi.nlm.nih.gov/geo/) under the following accession numbers: GSE202137 (RNA-seq), GSE205697 (ATAC-seq), GSE269913 (ATAC-seq), and GSE269910 (ACT-seq). Proteomics data are available via the PRIDE/ProteomeXchange repository under accession number PXD056378 (https://www.ebi.ac.uk/pride/archive/projects/PXD056378).
