# Supplementary material for: Role of MNX1-mediated histone modifications and PBX gene family in MNX1-induced leukemogenesis
Source: Sci Rep. 2026 Jan 19;16:2593. doi: 10.1038/s41598-026-36367-8 (PMC12820052; doi:10.1038/s41598-026-36367-8)
Supplement: Supplementary file 1 — Supplementary Material 1 [file 41598_2026_36367_MOESM1_ESM.docx]

**Supplementary Table S1. Sequences used in MNX1 plasmid constructs**

**Plasmid sequence**

# **HA-MNX1**

ATG*GAGGCCTATCCTTACGATGTGCCTGATTATGCATCTGGTAGTACTGCGCAGTCGACG*GAAAAGAGCAAGAACTTTAGAATAGACGCCCTTTTGGCTGTCGACCCACCTCGCGCAGCTAGTGCACAAAGCGCTCCTCTGGCCTTGGTTACATCCCTGGCTGCTGCCGCATCTGGAACCGGTGGCGGAGGCGGGGGCGGCGGAGCCTCTGGGGGGACTTCCGGTAGCTGTAGTCCTGCAAGTTCCGAACCACCAGCCGCCCCAGCCGACCGCCTGCGGGCCGAGAGCCCATCTCCACCAAGGCTGTTGGCTGCTCACTGTGCCTTGCTCCCAAAACCCGGATTCCTCGGCGCAGGCGGAGGAGGGGGCGGGACTGGAGGAGGACACGGAGGCCCTCATCACCATGCTCATCCTGGCGCAGCAGCTGCAGCAGCTGCTGCCGCAGCTGCCGCCGCAGCAGGGGGCCTGGCTCTGGGCCTGCACCCCGGTGGCGCTCAGGGCGGAGCTGGCCTGCCGGCTCAGGCCGCACTTTACGGCCACCCGGTCTATGGATACAGCGCTGCCGCCGCCGCCGCCGCCTTGGCTGGACAACACCCAGCACTTTCTTACAGTTATCCACAGGTGCAGGGAGCCCACCCGGCCCATCCCGCAGACCCTATTAAACTGGGGGCTGGGACGTTCCAGCTGGACCAGTGGCTCAGAGCATCCACTGCTGGGATGATCCTGCCCAAGATGCCTGACTTCAACAGTCAGGCTCAGTCAAACCTTCTGGGTAAGTGCCGACGACCACGGACAGCTTTCACATCACAGCAACTGTTGGAACTGGAGCACCAATTTAAGCTGAATAAGTACCTCTCCAGACCCAAGAGGTTCGAAGTTGCCACCAGCCTGATGCTTACTGAAACCCAAGTCAAGATATGGTTTCAGAACAGACGCATGAAGTGGAAGCGAAGTAAGAAGGCTAAGGAACAGGCCGCACAGGAGGCCGAAAAGCAGAAGGGGGGAGGCGGCGGTGCCGGTAAAGGCGGTGCCGAGGAGCCAGGAGCAGAGGAGCTGCTGGGCCCACCAGCTCCTGGCGACAAAGGGTCAGGGAGACGGCTCCGGGATCTCCGAGACAGCGATCCAGAGGAGGACGAGGACGAGGATGATGAAGATCACTTTCCCTACTCCAACGGGGCTAGTGTTCACGCAGCCAGCTCAGACTGTAGTAGCGAAGACGATTCCCCCCCACCTAGACCTTCTCACCAACCTGCCCCCCAATGA

*HA-tag*

*Linker region*
