# Supplementary material for: Role of MNX1-mediated histone modifications and PBX gene family in MNX1-induced leukemogenesis
Source: Sci Rep. 2026 Jan 19;16:2593. doi: 10.1038/s41598-026-36367-8 (PMC12820052; doi:10.1038/s41598-026-36367-8)
Supplement: Supplementary file 2 — Supplementary Material 2 [file 41598_2026_36367_MOESM2_ESM.docx]

**Supplementary Table S2. Primer sequences used for qPCR and ChIP-qPCR analysis of gene expression.**

**Primer sequences**

**Primers for SYBR assays (for codon optimized transcripts)**

| **Primer name** | **Sequence 5’-3’** |
| --- | --- |
| hMNX1ex2-3 fwd | GCT TAC TGA AAC CCA AGT CAA GA |
| hMNX1ex2-3 rev | TTC TGC TTT TCG GCC TCC T |

**TaqMan Gene Expression Assays from Applied Biosystems**

| **Gene** | **Assay** |
| --- | --- |
| mHprt (ref gene) | Mm01545399_m1 |
| mMnx1 | Mm01222622_m1 |
| mPbx1 | Mm04207617_m1 |
| mPbxIP1 | Mm05906812_s1 |
| mPbx4 | Mm00453088_m1 |

***Primers used for ChIP-qPCR***

| **Gene** | **Assay** |
| --- | --- |
| Pbx1-fwd | CCTACTTTCCACCCAGCGTC |
| Pbx1-rev | GTCATAGCTCCGCCTCCTTC |
| Random region- fwd | ACAAACAGCCCTCAAAAGCA |
| Random region -rev | AGCAAGGTAGCTCTGTGTTAGT |
