# Supplementary material for: Role of MNX1-mediated histone modifications and PBX gene family in MNX1-induced leukemogenesis
Source: Sci Rep. 2026 Jan 19;16:2593. doi: 10.1038/s41598-026-36367-8 (PMC12820052; doi:10.1038/s41598-026-36367-8)
Supplement: Supplementary file 8 — Supplementary Material 8 [file 41598_2026_36367_MOESM8_ESM.docx]

**Supplementary Table S8. TARGET patient case IDs and sample groups.**

| **TARGET CASE ID** | **Sample group** |  | **TARGET CASE ID** | **Sample group** |
| --- | --- | --- | --- | --- |
| TARGET-20-PARUNX | t(7;12) |  | TARGET-00-RO02395 | Normal BM |
| TARGET-20-PASIBG | t(7;12) |  | TARGET-00-RO02401 | Normal BM |
| TARGET-20-PAWBTJ | t(7;12) |  | TARGET-00-RO02417 | Normal BM |
| TARGET-20-PAVCJB | t(7;12) |  | TARGET-00-RO02431 | Normal BM |
| TARGET-20-PAWMUZ | t(7;12) |  | TARGET-00-RO02434 | Normal BM |
| TARGET-20-PAWNHH | t(7;12) |  | TARGET-00-RO02441 | Normal BM |
| TARGET-20-PAWNYK | t(7;12) |  | TARGET-00-RO02447 | Normal BM |
| TARGET-20-PAVXPB | t(7;12) |  | TARGET-00-RO02462 | Normal BM |
| TARGET-20-PAVXZL | t(7;12) |  | TARGET-00-RO02466 | Normal BM |
| TARGET-20-PAXEWS | t(7;12) |  | TARGET-00-RO02485 | Normal BM |
| TARGET-20-PAXHGR | t(7;12) |  | TARGET-00-RO02496 | Normal BM |
| TARGET-20-PAXMPG | t(7;12) |  | TARGET-00-RO02505 | Normal BM |
| TARGET-00-BM3897 | Normal BM |  | TARGET-00-RO02580 | Normal BM |
| TARGET-00-BM3969 | Normal BM |  | TARGET-00-RO02590 | Normal BM |
| TARGET-00-BM4203 | Normal BM |  | TARGET-00-RO02605 | Normal BM |
| TARGET-00-BM4404 | Normal BM |  | TARGET-00-RO02609 | Normal BM |
| TARGET-00-BM4473 | Normal BM |  | TARGET-00-RO02619 | Normal BM |
| TARGET-00-BM4508 | Normal BM |  | TARGET-00-RO02645 | Normal BM |
| TARGET-00-BM4616 | Normal BM |  | TARGET-00-RO02652 | Normal BM |
| TARGET-00-BM4641 | Normal BM |  | TARGET-00-RO02722 | Normal BM |
| TARGET-00-BM5682 | Normal BM |  | TARGET-00-RO02748 | Normal BM |
| TARGET-00-BM5751 | Normal BM |  | TARGET-00-RO02756 | Normal BM |
| TARGET-00-BM5756 | Normal BM |  | TARGET-00-RO02776 | Normal BM |
| TARGET-00-BM5759 | Normal BM |  | TARGET-00-RO02815 | Normal BM |
| TARGET-00-BM5776 | Normal BM |  | TARGET-20-PADZKD | inv(16) |
| TARGET-00-RO02176 | Normal BM |  | TARGET-20-PAEENN | inv(16) |
| TARGET-00-RO02177 | Normal BM |  | TARGET-20-PAEFGR | inv(16) |
| TARGET-00-RO02191 | Normal BM |  | TARGET-20-PAEGRE | inv(16) |
| TARGET-00-RO02201 | Normal BM |  | TARGET-20-PAKVYM | inv(16) |
| TARGET-00-RO02205 | Normal BM |  | TARGET-20-PALVKV | inv(16) |
| TARGET-00-RO02208 | Normal BM |  | TARGET-20-PANBYS | inv(16) |
| TARGET-00-RO02209 | Normal BM |  | TARGET-20-PANBZH | inv(16) |
| TARGET-00-RO02210 | Normal BM |  | TARGET-20-PANGCM | inv(16) |
| TARGET-00-RO02211 | Normal BM |  | TARGET-20-PANKKE | inv(16) |
| TARGET-00-RO02213 | Normal BM |  | TARGET-20-PANPTM | inv(16) |
| TARGET-00-RO02214 | Normal BM |  | TARGET-20-PANUTB | inv(16) |
| TARGET-00-RO02216 | Normal BM |  | TARGET-20-PANUUA | inv(16) |
| TARGET-00-RO02224 | Normal BM |  | TARGET-20-PANVGE | inv(16) |
| TARGET-00-RO02239 | Normal BM |  | TARGET-20-PANWHP | inv(16) |
| TARGET-00-RO02240 | Normal BM |  | TARGET-20-PANYGP | inv(16) |
| TARGET-00-RO02249 | Normal BM |  | TARGET-20-PANYNR | inv(16) |
| TARGET-00-RO02265 | Normal BM |  | TARGET-20-PAPAWN | inv(16) |
| TARGET-00-RO02280 | Normal BM |  | TARGET-20-PAPVCN | inv(16) |
| TARGET-00-RO02297 | Normal BM |  | TARGET-20-PAPVZK | inv(16) |
| TARGET-00-RO02327 | Normal BM |  | TARGET-20-PAPWYK | inv(16) |
| TARGET-00-RO02356 | Normal BM |  | TARGET-20-PAPXWI | inv(16) |
| TARGET-00-RO02361 | Normal BM |  | TARGET-20-PARBVE | inv(16) |
| TARGET-00-RO02379 | Normal BM |  | TARGET-20-PARCHW | inv(16) |
| TARGET-00-RO02380 | Normal BM |  | TARGET-20-PARDMG | inv(16) |
| TARGET-00-RO02391 | Normal BM |  | TARGET-20-PAREFM | inv(16) |

**Supplementary Table S8. Continuation.**

| **TARGET CASE ID** | **Sample group** |  | **TARGET CASE ID** | **Sample group** |
| --- | --- | --- | --- | --- |
| TARGET-20-PARHJV | inv(16) |  | TARGET-20-PASSSI | MLL |
| TARGET-20-PARHVI | inv(16) |  | TARGET-20-PASSWG | MLL |
| TARGET-20-PARIMT | inv(16) |  | TARGET-20-PASTUH | MLL |
| TARGET-20-PARJYP | inv(16) |  | TARGET-20-PASVVS | MLL |
| TARGET-20-PARLSW | inv(16) |  | TARGET-20-PASVYL | MLL |
| TARGET-20-PARMZF | inv(16) |  | TARGET-20-PASWAJ | MLL |
| TARGET-20-PARPWL | inv(16) |  | TARGET-20-PASWLN | MLL |
| TARGET-20-PARSHM | inv(16) |  | TARGET-20-PASWTY | MLL |
| TARGET-20-PARTAL | inv(16) |  | TARGET-20-PASZLJ | MLL |
| TARGET-20-PARUDL | inv(16) |  | TARGET-20-PATDNN | MLL |
| TARGET-20-PARXBT | inv(16) |  | TARGET-20-PATFDF | MLL |
| TARGET-20-PARYFN | inv(16) |  | TARGET-20-PATJHJ | MLL |
| TARGET-20-PASFJB | inv(16) |  | TARGET-21-PASIGA | MLL |
| TARGET-20-PASMHY | inv(16) |  | TARGET-21-PASSLT | MLL |
| TARGET-20-PASYJI | inv(16) |  | TARGET-21-PATAIJ | MLL |
| TARGET-20-PATIAK | inv(16) |  | TARGET-20-PABHET | t(8;21) |
| TARGET-20-PAEAKL | MLL |  | TARGET-20-PABHKY | t(8;21) |
| TARGET-20-PAEGRI | MLL |  | TARGET-20-PADDXZ | t(8;21) |
| TARGET-20-PAEIKD | MLL |  | TARGET-20-PAEJBT | t(8;21) |
| TARGET-20-PAKIYW | MLL |  | TARGET-20-PAERAH | t(8;21) |
| TARGET-20-PAKLPD | MLL |  | TARGET-20-PAKRUP | t(8;21) |
| TARGET-20-PAKTCX | MLL |  | TARGET-20-PALFVW | t(8;21) |
| TARGET-20-PANFMG | MLL |  | TARGET-20-PALGKX | t(8;21) |
| TARGET-20-PANPKN | MLL |  | TARGET-20-PANDER | t(8;21) |
| TARGET-20-PANZKA | MLL |  | TARGET-20-PANHYK | t(8;21) |
| TARGET-20-PAPWZR | MLL |  | TARGET-20-PANJGR | t(8;21) |
| TARGET-20-PAPZIZ | MLL |  | TARGET-20-PANLIZ | t(8;21) |
| TARGET-20-PARASV | MLL |  | TARGET-20-PANLRE | t(8;21) |
| TARGET-20-PARBIU | MLL |  | TARGET-20-PANNHB | t(8;21) |
| TARGET-20-PARBRA | MLL |  | TARGET-20-PANVGP | t(8;21) |
| TARGET-20-PARBXE | MLL |  | TARGET-20-PAPVDV | t(8;21) |
| TARGET-20-PARFAL | MLL |  | TARGET-20-PAPVGE | t(8;21) |
| TARGET-20-PARHVK | MLL |  | TARGET-20-PAPWHS | t(8;21) |
| TARGET-20-PARJCR | MLL |  | TARGET-20-PARBFZ | t(8;21) |
| TARGET-20-PARKCX | MLL |  | TARGET-20-PARCVS | t(8;21) |
| TARGET-20-PARKFB | MLL |  | TARGET-20-PARCZL | t(8;21) |
| TARGET-20-PARWDZ | MLL |  | TARGET-20-PARDDA | t(8;21) |
| TARGET-20-PARXZP | MLL |  | TARGET-20-PARENB | t(8;21) |
| TARGET-20-PASCGR | MLL |  | TARGET-20-PARGTL | t(8;21) |
| TARGET-20-PASDGX | MLL |  | TARGET-20-PARGVC | t(8;21) |
| TARGET-20-PASEFD | MLL |  | TARGET-20-PARIZR | t(8;21) |
| TARGET-20-PASFNP | MLL |  | TARGET-20-PARLMY | t(8;21) |
| TARGET-20-PASGGK | MLL |  | TARGET-20-PARTST | t(8;21) |
| TARGET-20-PASGZS | MLL |  | TARGET-20-PARURW | t(8;21) |
| TARGET-20-PASLSD | MLL |  | TARGET-20-PARUUB | t(8;21) |
| TARGET-20-PASMGW | MLL |  | TARGET-20-PARZUU | t(8;21) |
| TARGET-20-PASNIY | MLL |  | TARGET-20-PASBHI | t(8;21) |
| TARGET-20-PASPFE | MLL |  | TARGET-20-PASHBI | t(8;21) |
| TARGET-20-PANZKA | MLL |  | TARGET-20-PASHYZ | t(8;21) |
| TARGET-20-PAPWZR | MLL |  | TARGET-20-PASJEJ | t(8;21) |

**Supplementary Table S8. Continuation.**

| **TARGET CASE ID** | **Sample group** |  |  |  |
| --- | --- | --- | --- | --- |
| TARGET-20-PASLDL | t(8;21) |  |  |  |
| TARGET-20-PASPKE | t(8;21) |  |  |  |
| TARGET-20-PASPSV | t(8;21) |  |  |  |
| TARGET-20-PASPTW | t(8;21) |  |  |  |
| TARGET-20-PASREH | t(8;21) |  |  |  |
| TARGET-20-PASWPT | t(8;21) |  |  |  |
| TARGET-20-PASXYG | t(8;21) |  |  |  |
| TARGET-20-PATAST | t(8;21) |  |  |  |
| TARGET-20-PATDHA | t(8;21) |  |  |  |
| TARGET-21-PASLZE | t(8;21) |  |  |  |
|  |  |  |  |  |
|  |  |  |  |  |
|  |  |  |  |  |
|  |  |  |  |  |
|  |  |  |  |  |
|  |  |  |  |  |
|  |  |  |  |  |
|  |  |  |  |  |
|  |  |  |  |  |
|  |  |  |  |  |
|  |  |  |  |  |
|  |  |  |  |  |
|  |  |  |  |  |
|  |  |  |  |  |
|  |  |  |  |  |
|  |  |  |  |  |
|  |  |  |  |  |
|  |  |  |  |  |
|  |  |  |  |  |
|  |  |  |  |  |
|  |  |  |  |  |
|  |  |  |  |  |
|  |  |  |  |  |
|  |  |  |  |  |
|  |  |  |  |  |
|  |  |  |  |  |
|  |  |  |  |  |
|  |  |  |  |  |
|  |  |  |  |  |
|  |  |  |  |  |
|  |  |  |  |  |
|  |  |  |  |  |
|  |  |  |  |  |
|  |  |  |  |  |
|  |  |  |  |  |
|  |  |  |  |  |
|  |  |  |  |  |
|  |  |  |  |  |
|  |  |  |  |  |
|  |  |  |  |  |
