# Supplementary material for: Role of MNX1-mediated histone modifications and PBX gene family in MNX1-induced leukemogenesis
Source: Sci Rep. 2026 Jan 19;16:2593. doi: 10.1038/s41598-026-36367-8 (PMC12820052; doi:10.1038/s41598-026-36367-8)
Supplement: Supplementary file 11 — Supplementary Material 11 [file 41598_2026_36367_MOESM11_ESM.pdf]

Supplementary figure legends

Supplementary Figure 1. TMT-based Identification of MNX1-Associated Proteins. (A) Schematic diagram illustrating the sequential steps and analytical methods employed in the current study. Pre-leukemic stages are highlighted in light blue, leukemic stages in red**. (B)** Confirmation of immunoprecipitation was detected by western blot (WB) using HA antibodies after co-IP with HA magnetic beads between (M) and (C) samples. Actin was used as a loading control. Full uncropped WB images are provided in Supplementary Figure 13. **(C)** Heatmap of TMT-mass spectrometry quantification of MNX1 Co-IP comparing mice leukemia BM cells (M) with in vitro FL cells transduced with empty vector (C).

Supplementary Figure 2. Differential gene expression resulting from MNX1 ectopic expression in *in vitro* fetal liver cells compared to cells transduced with an empty vector. (A) Log fold change heat map of downregulated (blue) and upregulated (red) differentially expressed genes of FL cells transduced with MNX1 (M) in comparison with FL cells with empty vector (C). **(B)** Volcano plot depicting gene differential expression analysis of MNX1-vector transduced *in vitro* FL cells relative to empty vector transduced FL cells, (red) genes that are differentially expressed according to the cut-off criteria of a p-value<0.05 (log-transformed in the figure) and log2 fold change > |1|, (green) genes not differentially expressed only achieving log2 fold change > |1|, (blue) genes not differentially expressed only achieving a p-value<0.05, (gray) genes not differentially expressed.

Supplementary Figure 3. Transcription factors associated with MNX1-driven gene expression. (A-B) Barplot of normalized enrichment score (NES) for significant gene sets as determined by GSEA using the TF legacy gene set collections and the differentially expressed genes in **(A)** mice leukemia BM cells. **(B)** pre-transplantation invitro FL cells transduced with MNX1.

Supplementary Figure 4. Pbx family gene expression in MNX1 mouse model and across pediatric AML subtypes. (A) Box-and-whisker plots show median, interquartile range, and full data range of **(a)** Relative expression of *Pbx2*, and *Pbx3* normalized to *Hprt* and measured by qPCR in MNX1-transduced fetal-liver cells versus empty-vector control (Ctrl). DESeq2-normalized RNA-seq expression of PBX1, PBX2, PBX3, and MNX1 **(B)**, and PBX4 and PBXIP1 **(C)**, across pediatric AML cytogenetic subtypes including MLL, inv(16), and t(8;21), compared with t(7;12) pediatric AML samples and with normal bone marrow from healthy donors (TARGET AML dataset; Supplementary Table S8). Tukey's multiple comparisons test was used for post-hoc analysis. Significance is indicated as $p < 0.05$ (*), $p < 0.01$ (**), $p < 0.001$ (***), and $p < 0.0001$ (****).

Supplementary Figure 5. Alterations in histone methylation are associated with leukemia development. (A) GSEA enrichment plot for H3K4methylation in (upper) leukemia BM cells relative to *in vitro* FL cells and (lower) FL cells transduced with MNX1 vector relative to FL cells transduced with empty vector. **(B)** Heatmap depicting expression of H3K4methylation gene set in (left) FL cells transduced with MNX1 vector and (right) leukemia BM cells.

Supplementary Figure 6. Annotation and Heatmap Analysis of H3K4me3 and H3K4me1. (A) Heatmap of regions with differentially increased (green) and decreased (red) enrichment of

H3K4me3 in mice leukemia BM cells (LK) relative to FL cells transduced with empty vector (Set-Ctr), as well as a corresponding pie chart displays the annotation of these regions. **(B)** Heatmap of regions with differentially increased (green) and decreased (red) enrichment of H3K4me1 in mice leukemia BM cells (LK) relative to FL cells transduced with empty vector (Set-Ctr) as well as a corresponding pie chart displays the annotation of these regions. Regions with a log fold-change (logFC) ≥ |1| and false discovery rate (FDR) of ≤0.05 were considered differentially bound.

Supplementary Figure 7. Pathway enrichment analysis of promoter regions showing differential H3K4me3 enrichment in MNX1-induced leukemic bone marrow (BM) cells. Bubble plots summarize pathways associated with increased (A) and decreased (B) promoter-linked H3K4me3 enrichment in leukemic BM compared with MNX1-transduced in vitro fetal-liver (FL) cells. The analysis was performed on the aggregate set of differential promoter regions identified by ACT-seq using the ClusterProfiler package. Each bubble represents an enriched pathway; bubble size indicates the number of associated genes, and color reflects the adjusted *p*-value of enrichment. No individual replicates are shown, as the plots display combined enrichment results from the differential analysis rather than replicate-specific data. Full lists of all significantly enriched pathways are provided in the Supplementary Tables S9 and S10.

Supplementary Figure 8. Correlation analysis of samples similarity across ACT-seq and ATAC-seq data. (A-B) Jaccard statistic showing similarity between **(A)** ATAC peak-files, **(B)** H3K4me1 ACT-seq peak-files, **(C)** Jaccard statistic between H3K4me3 ACT-seq peak-files and **(D)** Jaccard statistic between MNX1-HA peak-files as determined by bedtools jaccard function. **(E)** Network graph showing the percentage of overlap between the replicates of MNX1 ACT-seq (nodes). LK1-B: MNX1 BM.1, LK2-B: MNX1 BM.2, LK3-B: MNX1 BM.3, LK4-B: MNX1 BM.4

Supplementary Figure 9. Comparative Analysis of Sample Similarity in MNX1 ACT-seq Data. (A-C) Heatmap displaying MNX1 binding profiles at transcription start sites (TSS) obtained from the refTSS database. Read counts from all MNX1 ACT-seq experiments were extracted within a ±1 kb window around the TSS. Only regions where MNX1 peaks were detected in at least one replicate are shown, while regions lacking MNX1 peaks in all replicates were omitted. The red-to-white gradient represents high-to-low read counts in the respective regions. **(D-E)** Genomic occupancy profiles of ACT-seq signals, highlighting regions with consistently well-defined enrichment peaks across all replicates (right panel) extracted from **(C)** and regions exhibiting dynamic changes in peak enrichment between replicates (left panel) extracted from **(B).**

Supplementary Figure 10. Correlation analysis of MNX1-motifs. Lower half of a correlation matrix depicting the pairwise correlation between each of the top 10 motifs found in the MNX1 ACT-seq peaks of leukemic bone marrow cells from each individual mouse and in MNX1 ChIP-seq from insulinoma cells. The first of the two numbers indexing the motifs is the rank of the motif in the used peak set, and the second number denotes which peak set. 1=MNX1 BM1, 2=MNX1 BM2, 3=MNX1 BM3, 4=MNX1 BM4 and Ins=ChIP-seq of MNX1 in insulinoma cells. The correlation score is the average Pearson correlation of all possible pattern matches between two motifs. Clusters highlighted with a thick border are motifs found in all peak sets and the corresponding motif-logo is taken from the MNX1 BM1 peak set.

Supplementary Figure 11. Canonical MNX1 motif enrichment across MNX1-associated chromatin regions. (A) Bar plots showing the enrichment frequency of the canonical MNX1 motifs (blue)—ATTTA (upper panel) and TAATTA (lower panel)—previously identified using the Genomatix platform, compared with GC-matched randomized control sequences (orange). Motif enrichment was assessed within MNX1 ACT-seq peaks and in the public MNX1 ChIP-seq dataset GSE61432. **(B)** Bar plots showing enrichment of the same canonical MNX1 motifs (blue) versus GC-matched randomized sequences (orange) across ATAC-seq peaks, H3K4me1, and H3K4me3 regions.

Supplementary Figure 12. Pathway enrichment analysis for Pbx1 motifs enriched over MNX1 promoters. Bubble plot showing the pathway enrichment analysis for Pbx1 motifs enriched over MNX1 promoters from (upper) ATAC-seq and (lower) H3K4me3 ACT-seq, generated using the ClusterProfiler package. The size of each bubble corresponds to the gene count associated with the respective pathway, while the color gradient represents the adjusted p-value of the enriched pathways.

Supplementary Figure 13. Validation and source data for MNX1 co-immunoprecipitation. Uncropped Western blot images corresponding to Supplementary Figure 1 (B).

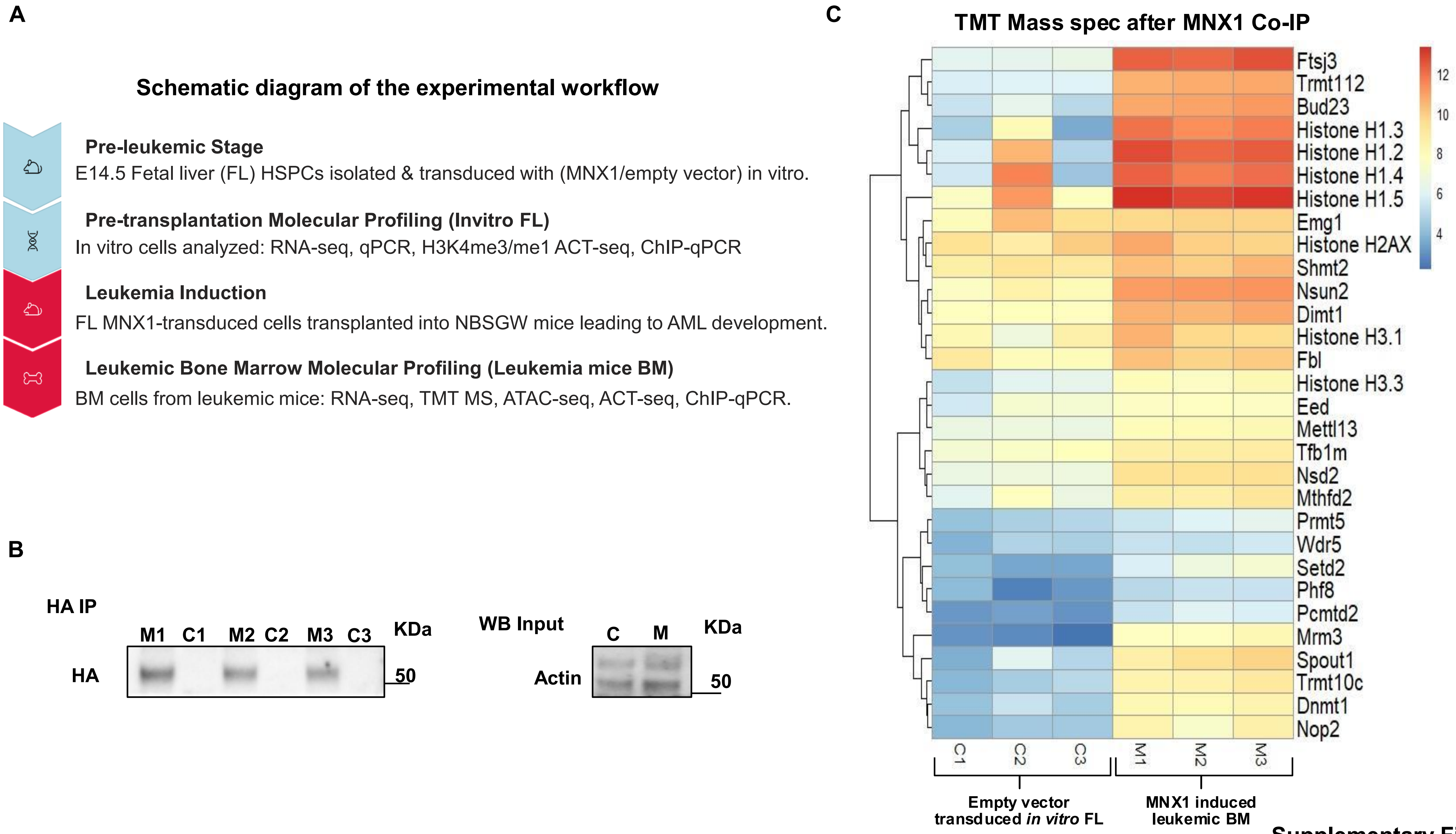

Supplementary Figure 1

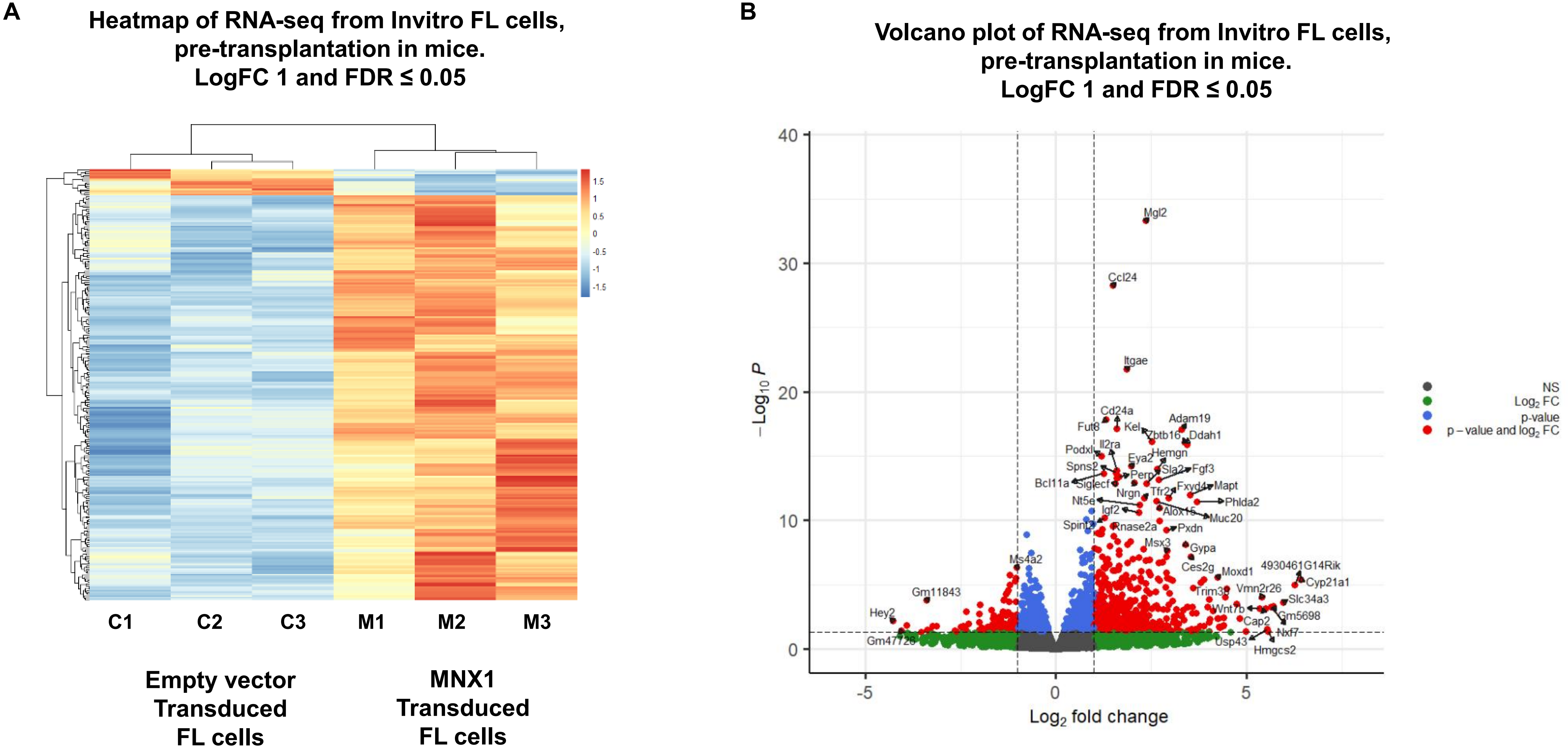

Supplementary Figure 2

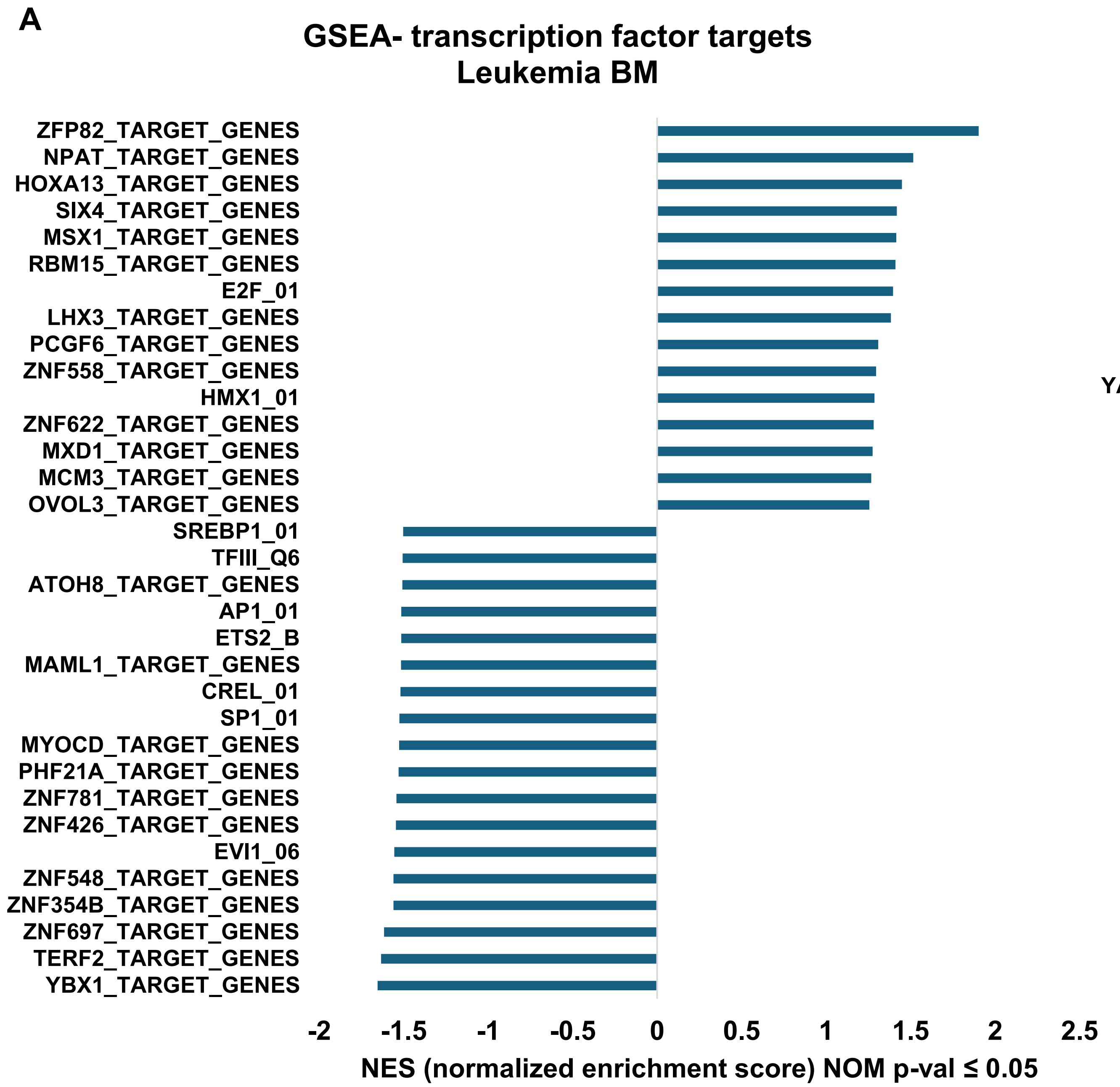

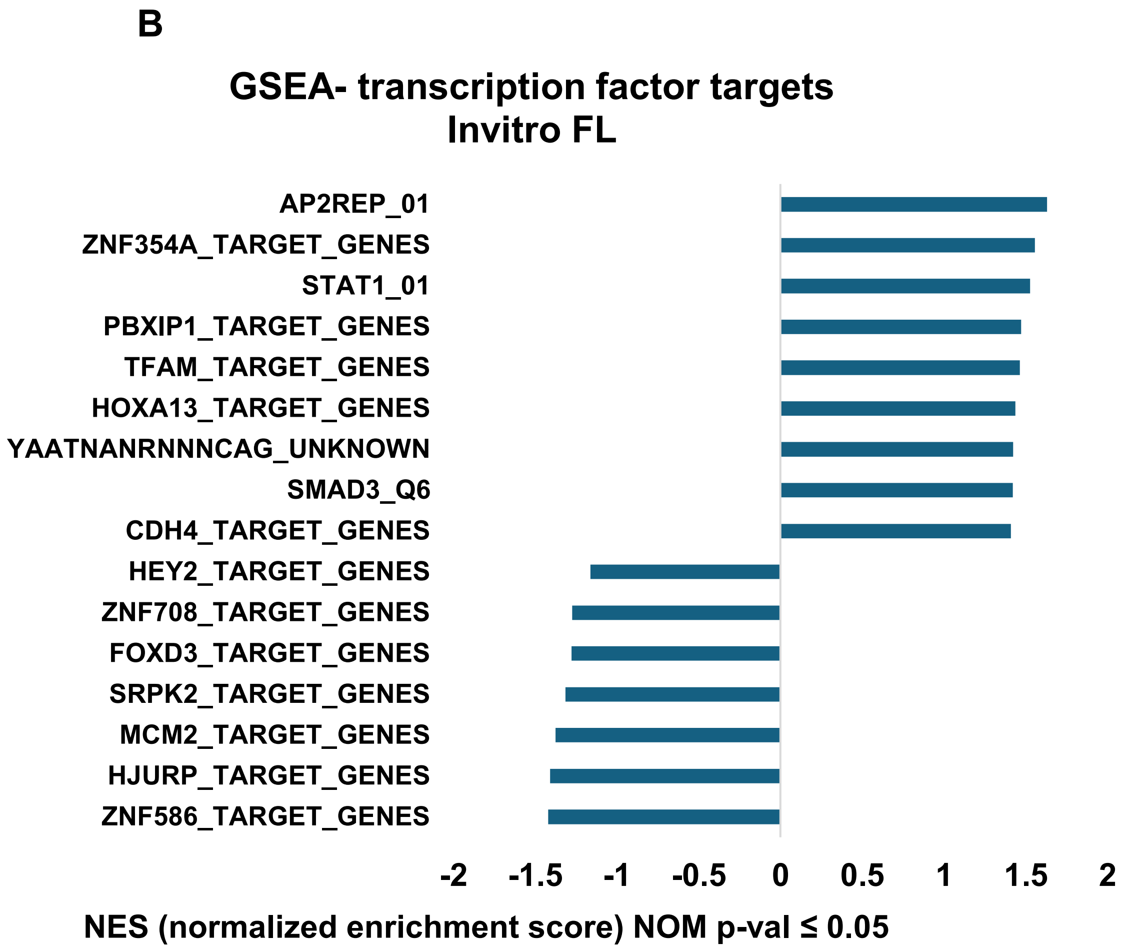

Supplementary Figure 3

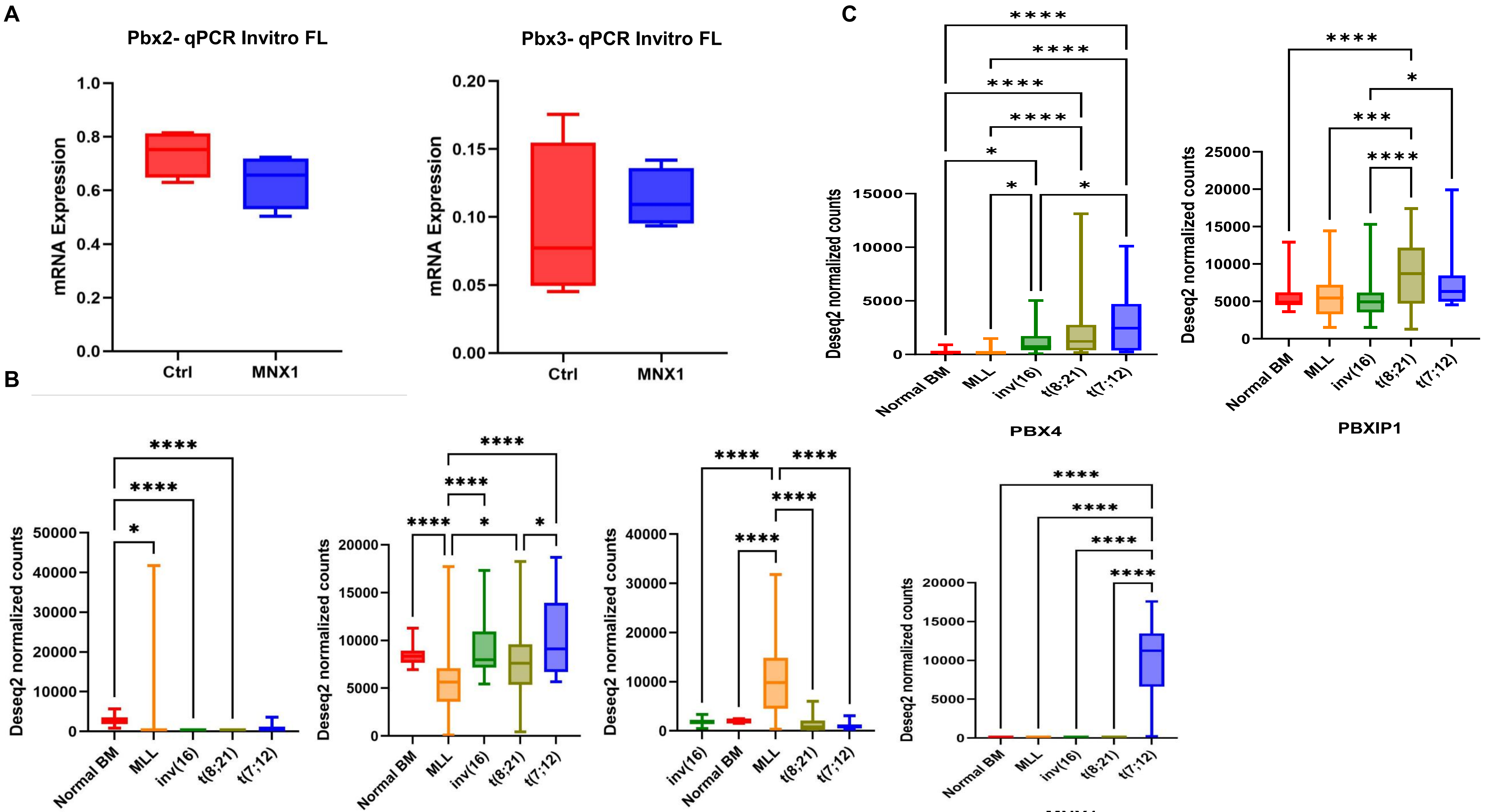

Supplementary Figure 4

A

In vivo BM from NSG mice

Leukemic BM vs Empty vector transduced FL cells

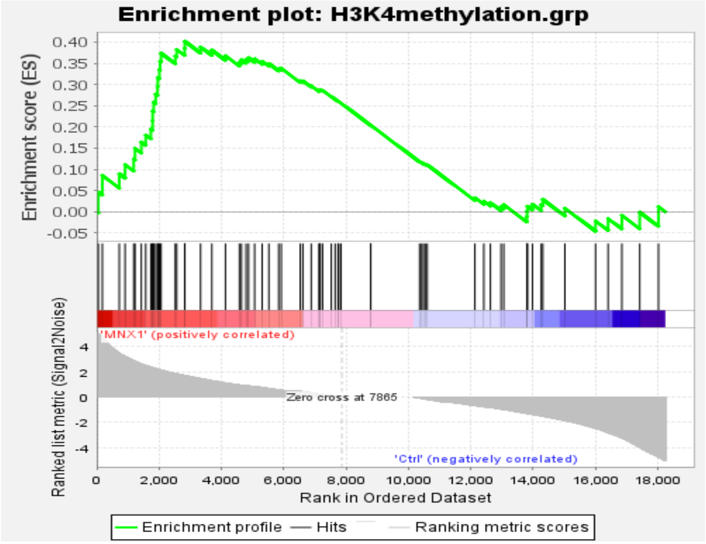

NES= 1.32
Nominal p-value = 0.0
FDR q-value = 0.0853
FWER p-value = 0.0

In vitro FL cells, pre-transplantation in mice

MNX1 transduced FL vs
Empty vector transduced FL cells

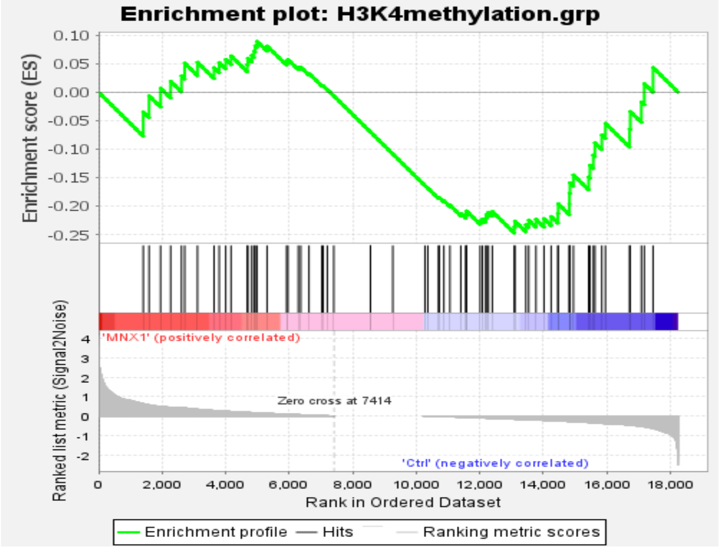

NES = -0.9
Nominal p-value = 0.38
FDR q-value = 0.49
FWER p-value = 0.194

B

In vitro FL cells

In vivo BM from NSG mice

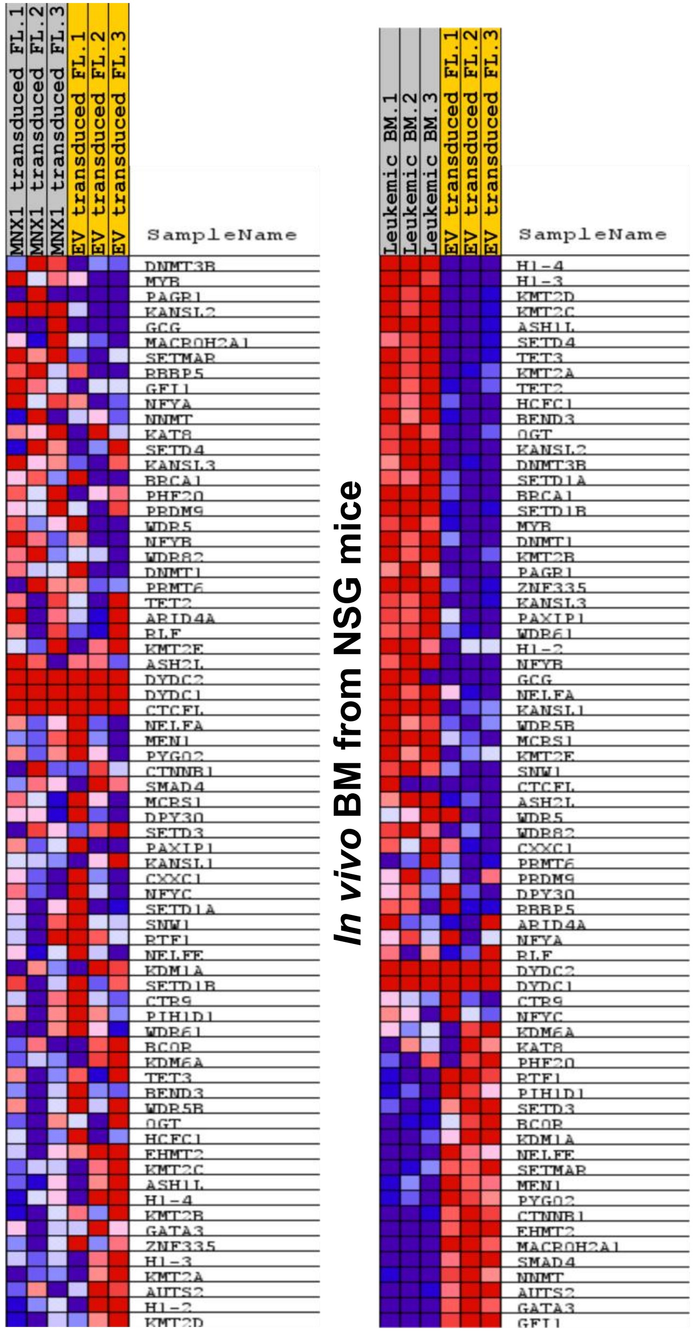

Supplementary Figure 5

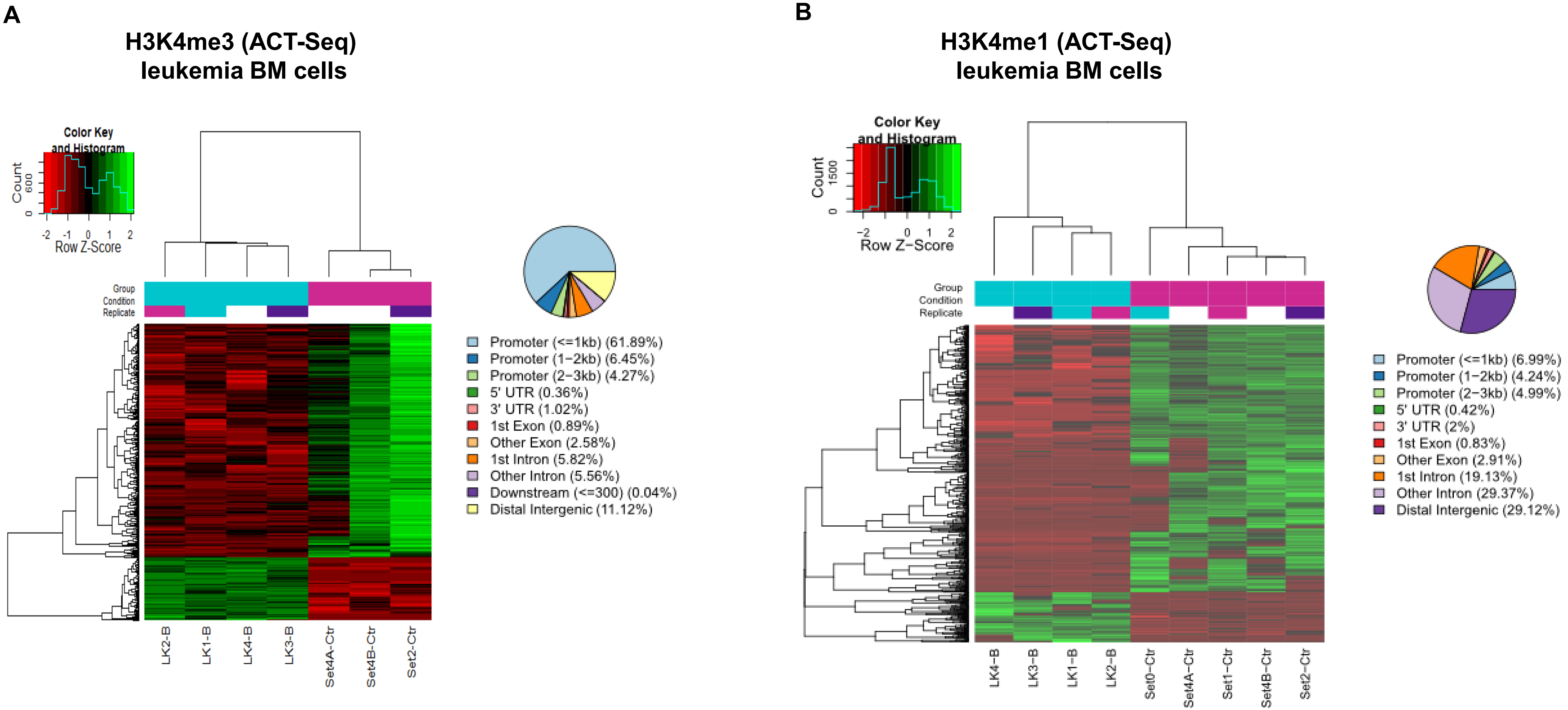

Supplementary Figure 6

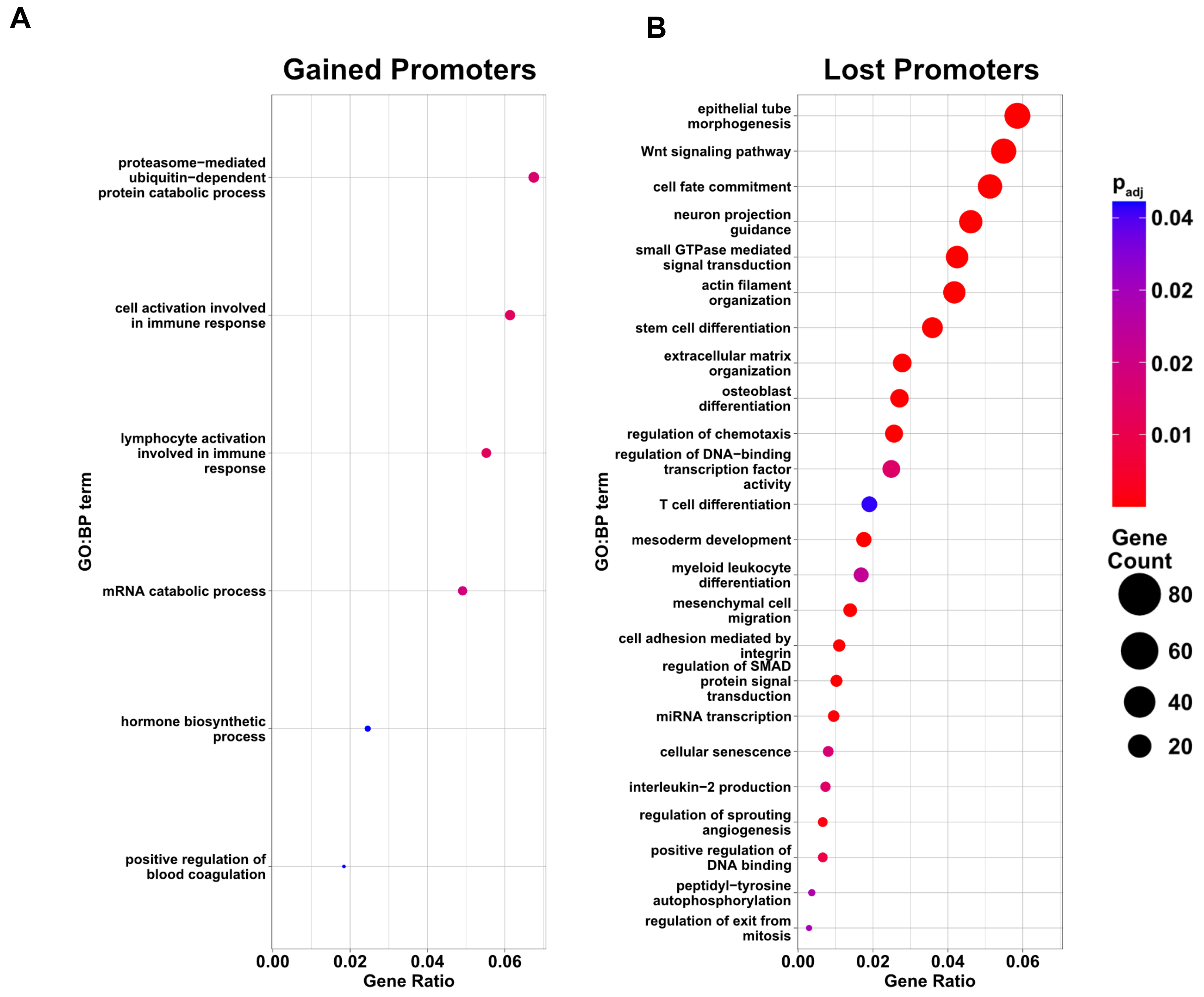

Supplementary Figure 7

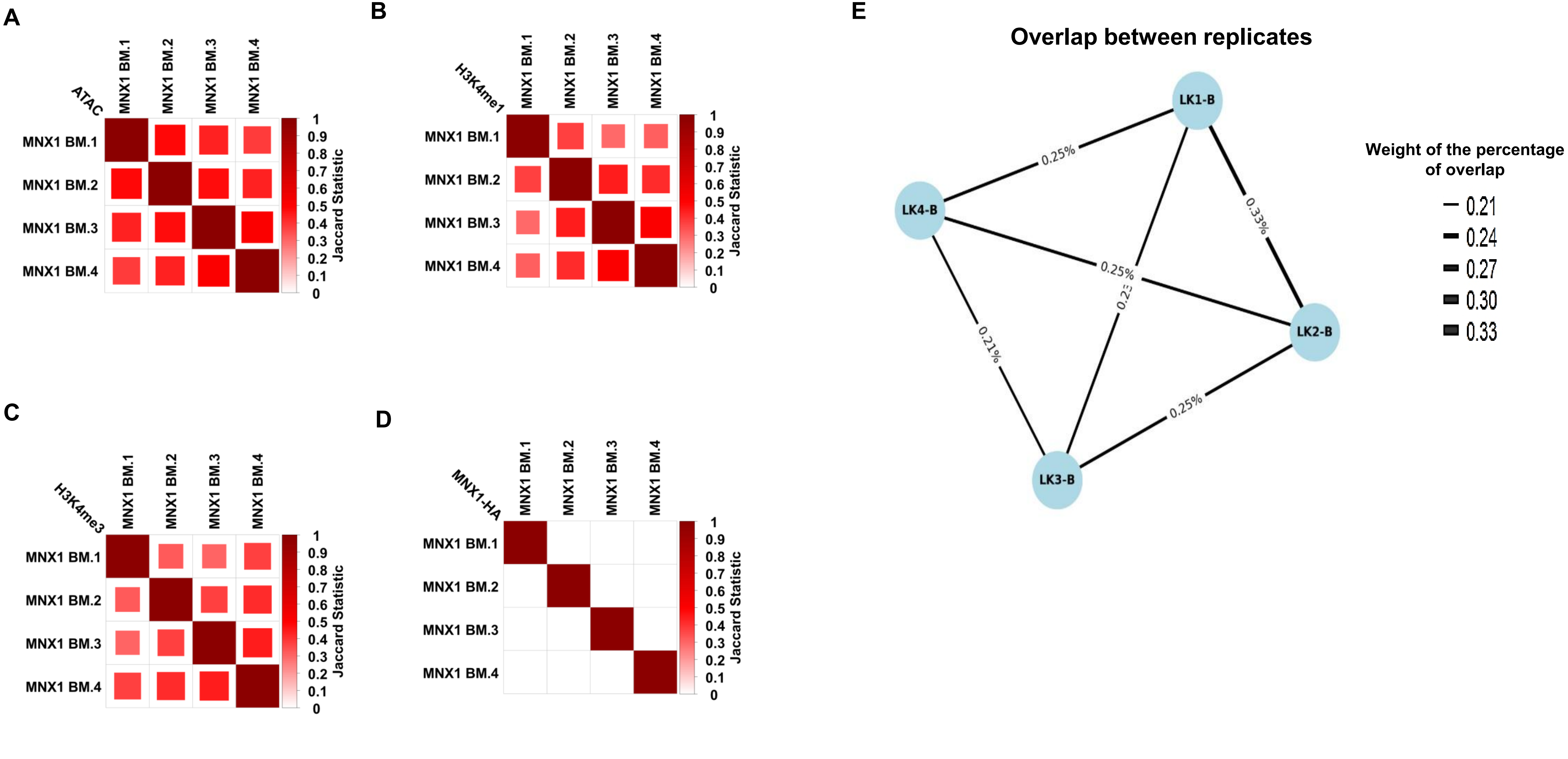

Supplementary Figure 8

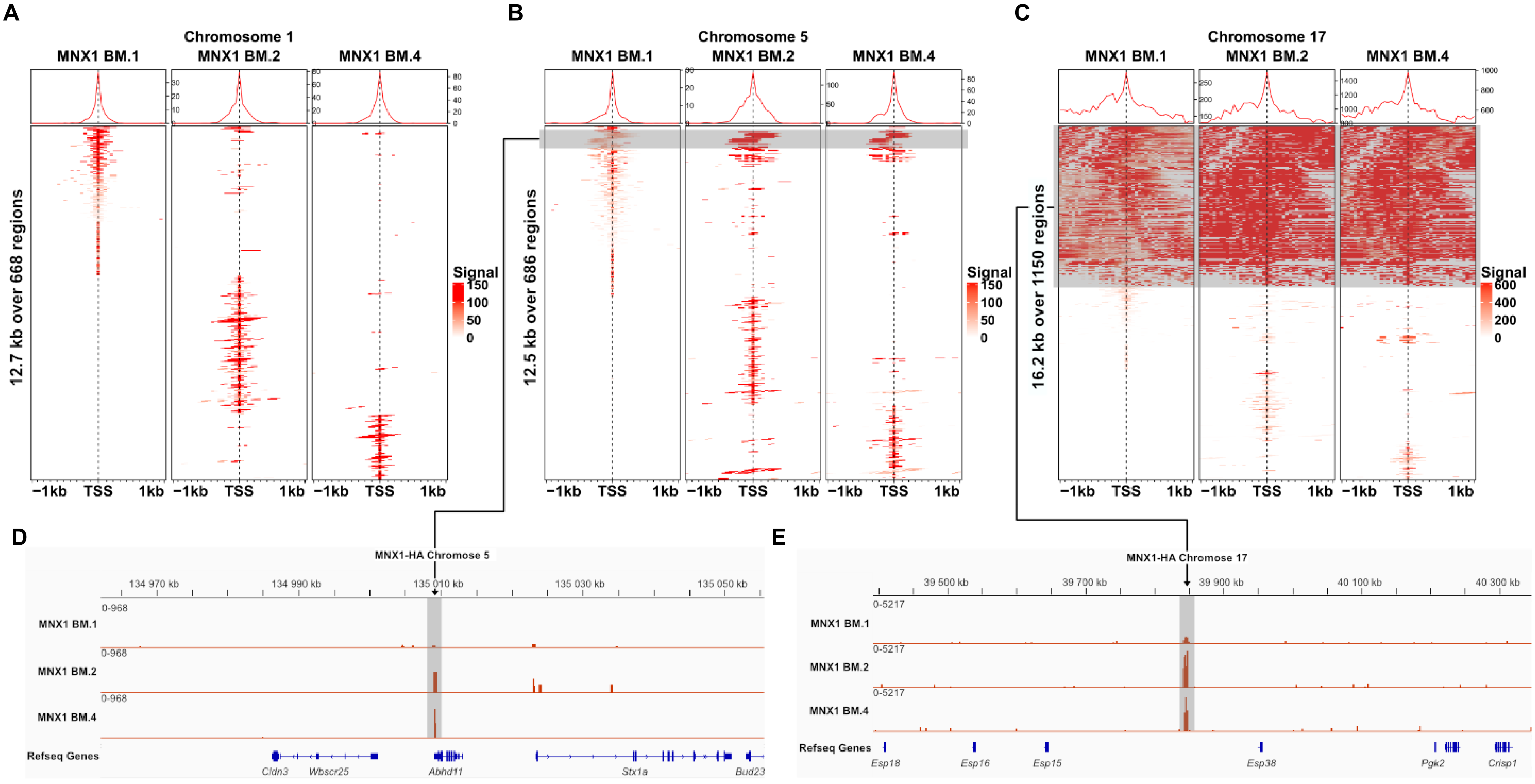

Supplementary Figure 9

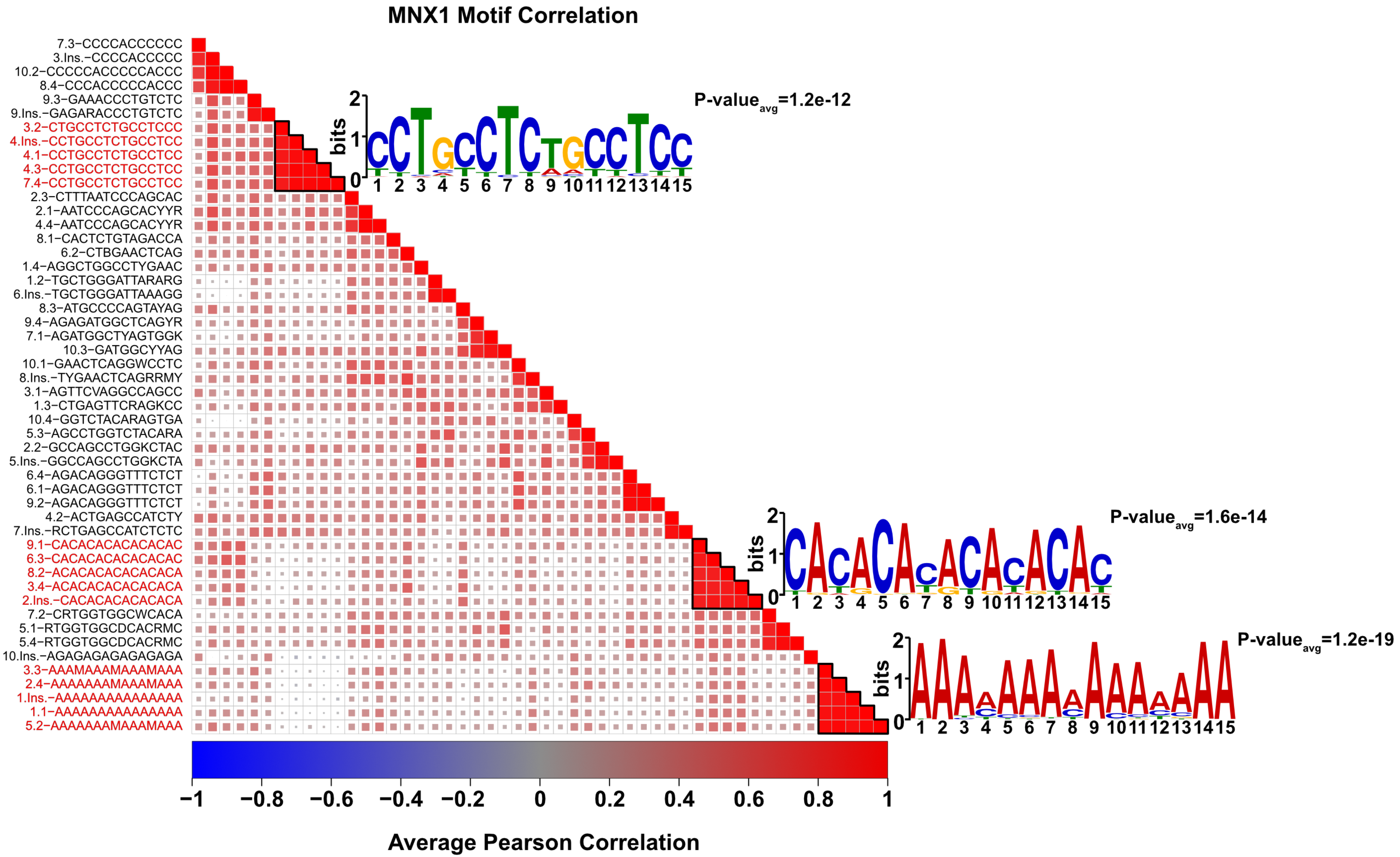

Supplementary Figure 10

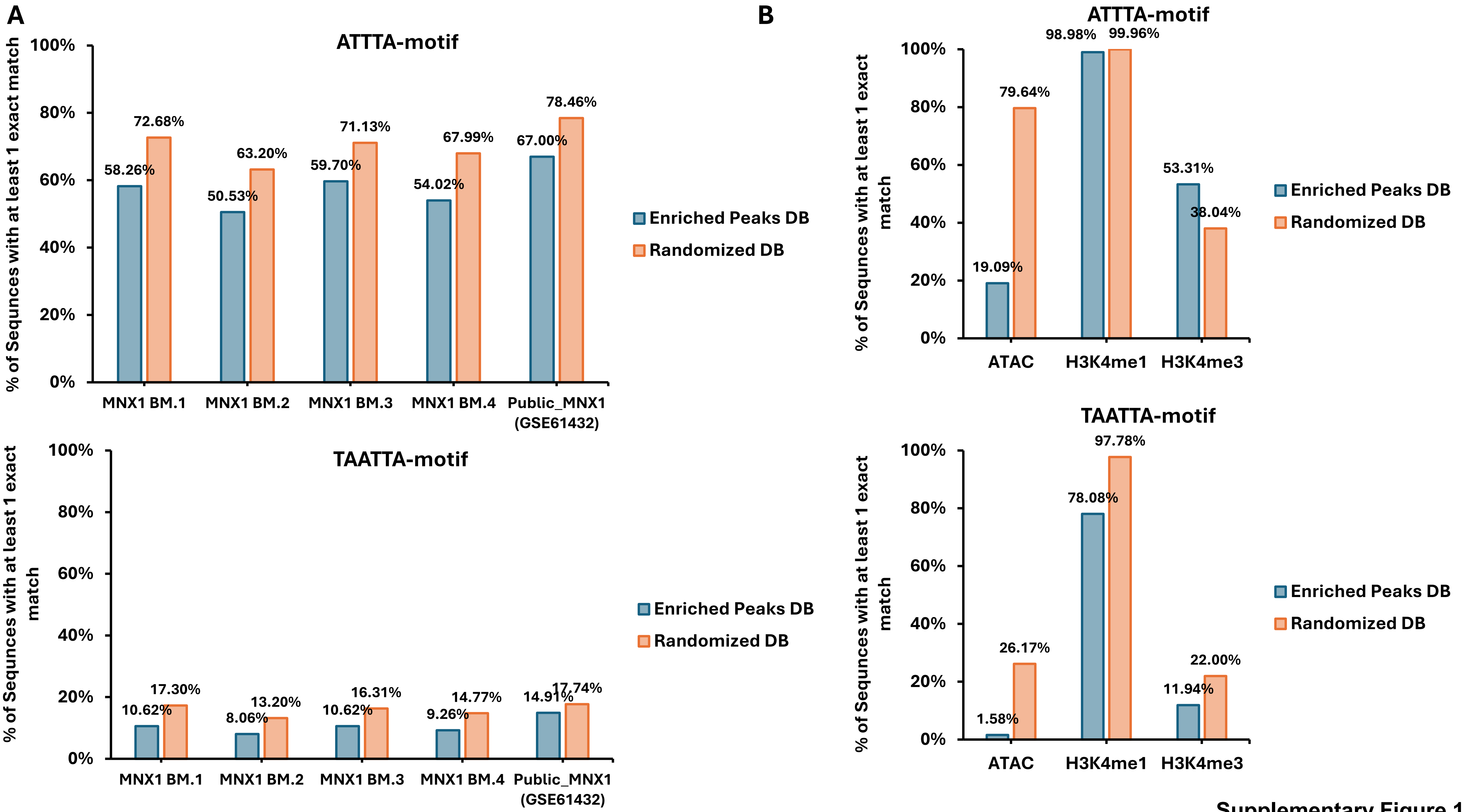

Supplementary Figure 11

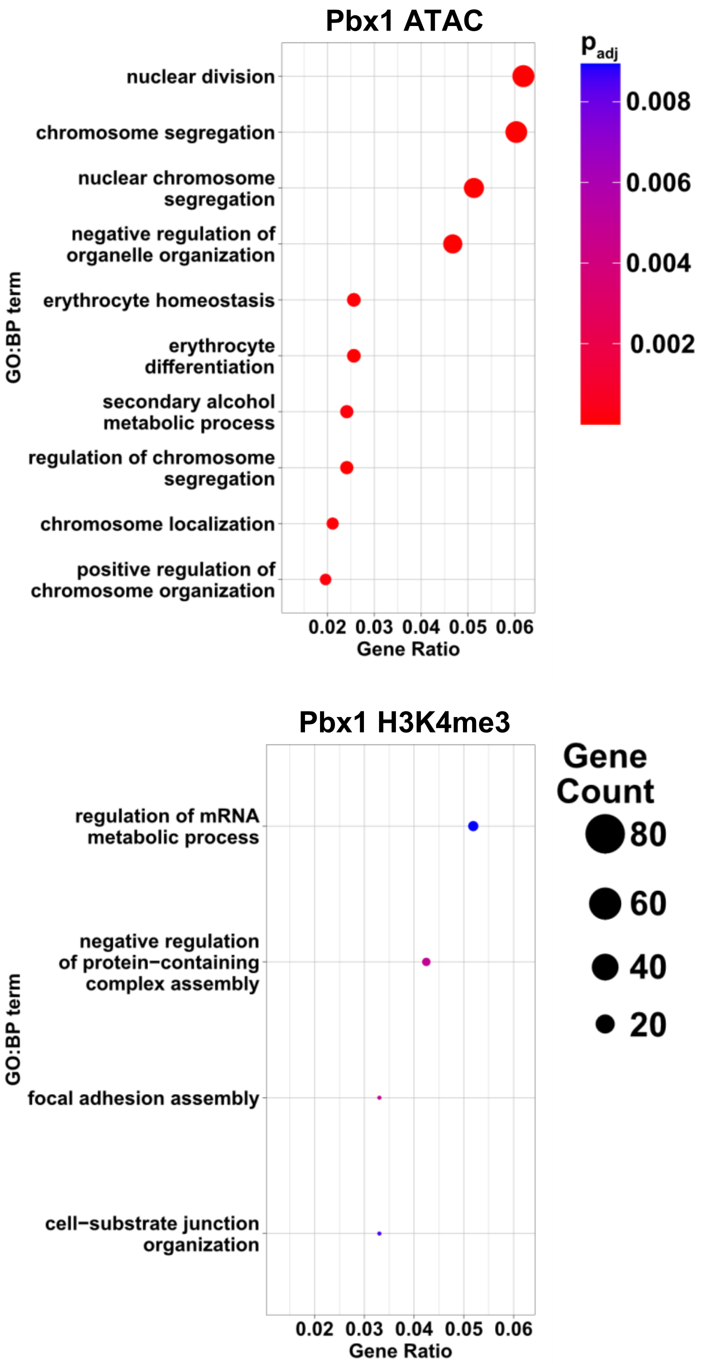

Supplementary Figure 12

A

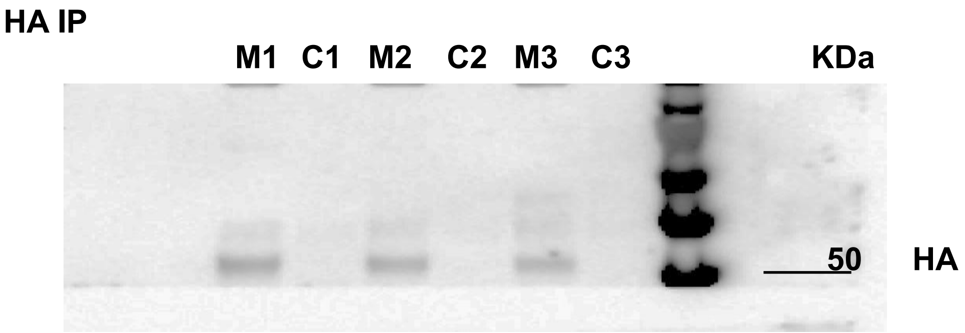

B

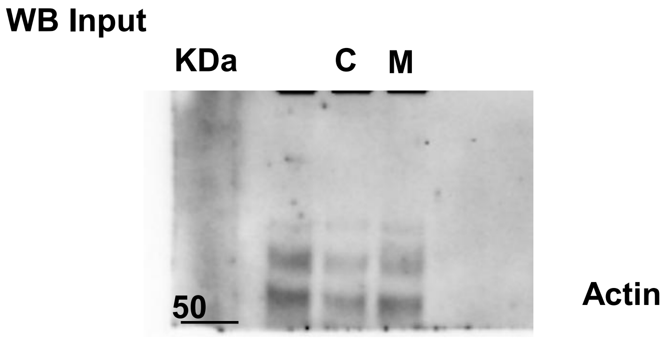

Supplementary Figure 13
